# Dual functions of Rap1 are crucial for T-cell homeostasis and prevention of spontaneous colitis

**DOI:** 10.1038/ncomms9982

**Published:** 2015-12-04

**Authors:** Sayaka Ishihara, Akihiko Nishikimi, Eiji Umemoto, Masayuki Miyasaka, Makoto Saegusa, Koko Katagiri

**Affiliations:** 1 Department of Biosciences, School of Science, Kitasato University, 1-15-1 Kitasato, Minamiku, Sagamihara, Kanagawa 252-0373, Japan; 2 Department of Microbiology and Immunology, Laboratory of Immune Regulation, Osaka University Graduate School of Medicine, 2-2 Yamadaoka, Suita, Osaka 565-0871, Japan; 3 Interdisciplinary Program for Biomedical Sciences, Institute for Academic Initiatives, Osaka University, 2-2 Yamadaoka, Suita, Osaka 565-0871, Japan; 4 Department of Pathology, School of Medicine, Kitasato University, 1-15-1 Kitasato, Minamiku, Sagamihara, Kanagawa 252-0373, Japan

## Abstract

Rap1-GTP activates leukocyte function-associated antigen-1 (LFA-1) to induce arrest on the high endothelial venule (HEV). Here we show that Rap1-GDP restrains rolling behaviours of T cells on the peripheral lymph node addressin (PNAd), P-selectin and mucosal addressin cell adhesion molecule-1 (MadCAM-1) by inhibiting tether formation. Consequently, Rap1 deficiency impairs homing of naive T cells to peripheral lymph nodes, but accelerates homing of T_H_17 and T_H_1 cells to the colon, resulting in spontaneous colitis with tumours. Rap1-GDP associates with and activates lymphocyte-oriented kinase, which phosphorylates ERM (ezrin, radixin and moesin) in resting T cells. Phosphomimetic ezrin reduces the rolling of Rap1-deficient cells, and thereby decreases their homing into the colon. On the other hand, chemokines activate Rap1 at the plasma membrane within seconds, and Rap1-GTP binds to filamins, which diminishes its association with the β_2_ chain of LFA-1 and results in LFA-1 activation. This Rap1-dependent regulation of T-cell circulation prevents the onset of colitis.

## Abstract (web summary)

Rap1, a member of the Ras family of small guanine triphosphatases, mediates lymphocyte adhesion to high endothelial venules. Here the authors show that depending on its activation status Rap1 plays a dual role in T cell adhesion and by regulating T cell homeostasis is involved in the protection from colitis.

## Graphical abstract

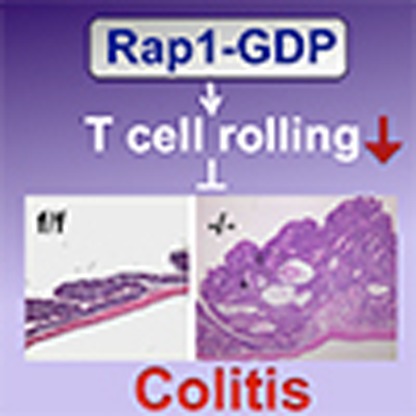

Lymphocytes recirculate continually between the peripheral lymphoid tissues via the blood and lymphatic systems^1^,^2^. Lymphocytes enter across the high endothelial venule (HEV) into lymphoid tissues via a specialized interaction with venule. Naive lymphocytes (T_N_) are first captured, and then they undergo rolling because of weak binding between L-selectin and sulfated sialyl Le^x^-related O-glycans expressed on HEVs, collectively called peripheral lymph node addressin (PNAd). When rolling lymphocytes are exposed to chemokines present on the HEV, chemokine signalling coupled with G_i_ proteins activates leukocyte function-associated-1 (LFA-1), a major receptor that mediates homing to peripheral lymph nodes. In a previous study, we showed that the small GTPase Rap1, which is rapidly activated by chemokines, is indispensable for LFA-1-dependent adhesion to the HEV^3^,^4^. LFA-1-dependent adhesion can be divided into several sequential steps: the RAPL–Mst1 complex, a downstream effector of Rap1, is involved in the stabilization step but not in the preceding LFA-1 activation step^5^,^6^. Therefore, the molecular mechanism of Rap1-dependent LFA-1 activation remains unsolved.

Activation of integrins is regulated by interactions with various intracellular adaptor proteins^7^. Cytoplasmic actin-binding proteins, such as talin, kindlin and filamin (FLN), bind directly to integrin tails and positively or negatively regulate integrin function: the currently available evidence indicates that talin and kindlin promote integrin activation, whereas FLN is a negative regulator of integrin functions, such as cell adhesion and migration^8^,^9^. FLN also serves as a scaffolding protein for Rho or Ras family members^10^. Since the deletion of cytoplasmic region of β_2_ induced spontaneous arrest^4^, the dissociation of a *trans*-acting restraint from the β_2_ cytoplasmic region of LFA-1 might be critical for Rap1-dependent lymphocyte arrest by chemokine.

CD4^+^ effector/memory T (T_EM_) cells are primarily found in the lamina propria (LP) of the intestine, and they play important roles in intestinal homeostasis. T_EM_ cells that populate the intestine are produced in mesenteric lymph nodes (mLNs) and Peyer's patches (PPs) and they move through draining lymphatic ducts, migrate into the bloodstream through the thoracic ducts and ultimately accumulate in the intestine. The integrin intestinal homing receptor α_4_*β*_7_ is required for trafficking of CD4^+^ T cells to the intestine and the induction and perpetuation of chronic colitis^11^. In the multistep leukocyte adhesion cascade, it is generally selectins that mediate rolling and integrins that mediate subsequent arrest. α_4_*β*_7_ clearly mediates rolling as well as firm adhesion *in vivo*^12^. Therefore, the efficiency of rolling directly reflects the ability of the cells to move to the mucosal tissues of the intestine. Mucosal addressin cell adhesion molecule-1 (MadCAM-1), a ligand for α_4_*β*_7_, is constitutively expressed in postcapillary venules of intestinal LP and acts as a key intestinal addressin for intestinal homing^13^. Blockade of MadCAM-1 depletes T_EM_ cells from the intestinal LP. Unlike T_N_ cells, T_EM_ cells express high levels of integrins and exhibit spontaneous binding to their ligands under shear flow in the absence of chemokine^14^. In addition, a previous study demonstrated that Rap1 is indispensable for LFA-1, but dispensable for α_4_ integrin-dependent adhesion^15^,^16^. The precise roles of Rap1 in intestinal homing of T_EM_ cells remain unknown.

Ezrin, radixin and moesin (ERM) proteins are important for the formation of blebs during cell polarization and migration^17^. The active form of ezrin increases membrane tension and impedes homing into lymph nodes in mice^18^. The plasma membrane/actin cytoskeleton crosslinking function of ERM proteins relies heavily on threonine phosphorylation-dependent changes in conformation. In contrast to other cells such as epithelial cells, ERM proteins in resting lymphocytes exist predominantly in their active (phosphorylated) conformation^17^. The active and the dormant conformations of ERMs are maintained by a balance of kinase and phosphatase activities. The lymphocyte-oriented kinase (LOK) phosphorylates ERM in resting lymphocytes^19^.

In this study, we dissects the regulatory mechanisms of the lymphocyte adhesion cascade by Rap1 and the roles of Rap1 *in vivo* using mice harbouring T-cell-specific knockouts of *Rap1a* and *Rap1b*. Rap1-GDP in resting T_N_ and T_EM_ cells retains cell rigidity and limits rolling behaviours in blood vessels, which retards lymphocyte homing. On the other hand, integrin activation by Rap1-GTP is an exclusive component of T_N_ cell recirculation. Therefore, Rap1 deficiency leads to lymphopenia subsequent a generation of pathogenic T_EM_ cells in LNs, and facilitates homing of T_EM_ cells into the colon, which exacerbates spontaneous T-cell-dependent colitis with tubular adenomas in the presence of regulatory T cells (Treg). Thus, regulation of T-cell trafficking by both Rap1-GTP and -GDP is a key control mechanism of mucosal tolerance.

## Results

### Rap1 deficiency facilitated L-selectin-dependent rolling

**Figure 1:**
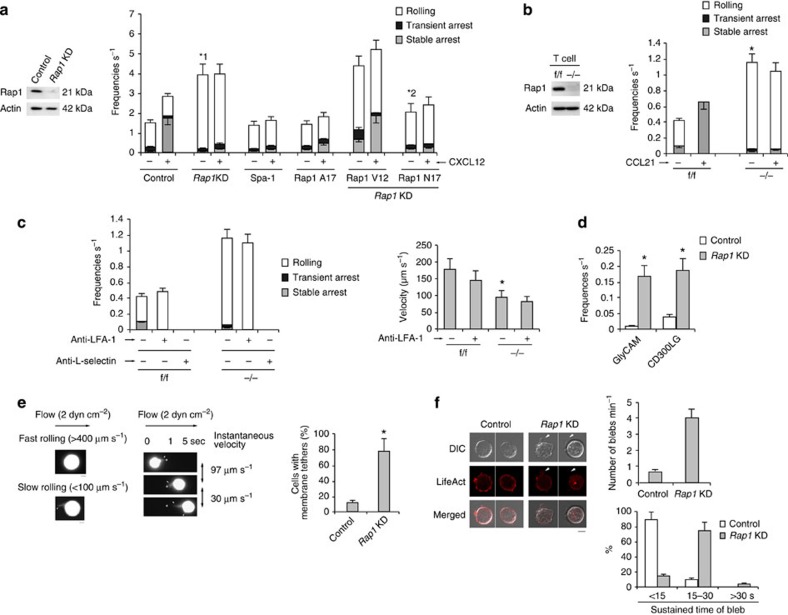
Rap1 deficiency enhances L-selectin-dependent rolling. (**a**; Left) Total lysates from BAF/LFA-1/L-selectin cells transduced with scramble (Control) or *Rap1a* or *b* (*Rap1*KD)-targeting short hairpin RNA (shRNA) were immunoblotted with anti-Rap1. Actin is a loading control. (Right) Control, *Rap1*KD, Spa-1or Rap1A17-expressing cells or *Rap1*KD cells transfected with Rap1V12 or Rap1N17 were perfused on LS12 monolayers immobilized ±100 nM CXCL12. Adhesive events of more than 100 cells were measured. *^1^*P*<0.001, *^2^*P*<0.006, relative to the total frequency of control cells or *Rap1*KD cells. (**b**; Left) Total lysates from *Rap1a*, *b*^f/f^ or *Rap1a*, *b*^−/−^ T cells were immunoblotted with anti-Rap1. Actin is a loading control. (Right) Adhesion of f/f or −/− T cells to LS12 cells expressing mouse ICAM-1 ±100 nM CCL21 was measured (*n*=100 of each group). *P<0.001 versus total frequency of f/f T cells. (**c**; Left) Effects of anti-LFA-1 or L-selectin on the interaction frequencies of f/f or −/− T cells on LS12 cells in the absence of CCL21. (Right) Rolling velocities of f/f or −/− T cells pre-treated ± anti–LFA-1 in the absence of CCL21 (*n*=100 of each group). *P<0.003 versus f/f cells. (**d**) Control or *Rap1*KD cells were perfused on plates immobilized with purified GlyCAM-1 or CD300LG (*n*=50 of each group). *^1^*P*<0.001 versus control cells. (**e**; Left) Morphology of rolling cells on the endothelium. A representative control cell exhibiting fast rolling and a *Rap1*KD cell exhibiting slow rolling are shown. Scale bars, 5 μm. (Centre) Sequential images of slow-rolling *Rap1*KD cell at the indicated times. Scale bars, 5 μm. Arrows show tethers. (Right) Ratios of cells exhibiting membrane tethers (*n*=50 cells of each group). *P<0.001 versus control cells. (**f**; Left) Confocal microscopic analysis of control or *Rap1*KD cells, transfected with LifeAct-mCherry. Scale bar, 5 μm. Arrows show blebs. (Right) Percentages of the cells showing number of blebs per minute (upper) and sustained bleb times (lower) in control or *Rap1*KD cells (*n*=50 of each group) are shown. All data show the means±s.e.m. or representative of three independent experiments.

Previously, we reconstituted the lymphocyte adhesion cascade using a BAF pro-B cell line expressing human L-selectin and LFA-1 (BAF/LFA-1/L-selectin)^4^. To examine the roles of Rap1 in this system, we knocked down Rap1 in BAF/LFA-1/L-selectin (control) cells. Protein levels in *Rap1a* and *Rap1b* knockdown (*Rap1*KD) cells were reduced to ∼20% of that in the control cells (Fig. 1a). As previously reported^4^, deletion of Rap1 inhibited CXCL12-induced arrest, which was mediated by LFA-1 (Fig. 1a). Unexpectedly, the frequencies of rolling on the endothelium were significantly elevated in *Rap1*KD cells in the absence of CXCL12 (Fig. 1a and Supplementary Movies 1 and 2). Overexpression of Spa-1, a Rap1-specific GTPase-activating protein or the dominant-negative form of Rap1 (Rap1A17)^20^, inhibited CXCL12-dependent arrest but did not increase the incidence of rolling (Fig. 1a), suggesting that loss of Rap1-GDP promotes the rolling behaviour. Consistent with these data, expression of a constitutively active mutant, Rap1V12, in *Rap1*KD cells did not suppress the frequency of rolling, whereas constitutively negative mutant, Rap1N17, reduced it (Fig. 1a and Supplementary Fig. 1a), suggesting that conversion of GDP to GTP promotes capture and subsequent rolling.

We examined Rap1 function in the lymphocyte adhesion cascade using primary mouse T cells. To generate *Rap1a* and *Rap1b* conditional double-knockout mice (Rap1 CKO), mice carrying floxed *Rap1a* and *b* alleles (*Rap1*^f/f^) were mated with lck-Cre or CD4-Cre transgenic mice to delete Rap1 specifically in T cells (*Rap1*^−/−^). Western blot analysis confirmed that the Rap1 protein was not expressed in T cells derived from those mice (Fig. 1b). *Rap1*^−/−^ T cells exhibited defects in chemokine-induced stable arrest on the endothelium, but a significantly elevated frequency of rolling in the absence of CCL21, relative to *Rap1*^f/f^ T cells (Fig. 1b and Supplementary Movies 3 and 4). Expression of LFA-1 and CCR7 did not significantly differ between f/f and −/− T cells (Supplementary Fig. 1b,c).

Anti-LFA-1 antibody did not affect the incidence of rolling in *Rap1*KD cells or *Rap1*^−/−^ T cells, but anti-L-selectin completely prevented rolling in both types of cells, suggesting that ablation of Rap1 promoted L-selectin-dependent rolling (Fig. 1c and Supplementary Fig.1d). Rolling velocity was reduced in *Rap1*KD and *Rap1*^−/−^ T cells, relative to the corresponding control cells, and entirely unaffected by anti-LFA-1 treatment (Fig. 1c and Supplementary Fig. 1d).

To verify the dependency on L-selectin, we infused control and *Rap1*KD cells over purified glycosylation-dependent cell adhesion molecule (GlyCAM) or Nepmucin (CD300LG)^21^. In *Rap1*KD cells, the frequency of rolling was increased more than 10-fold relative to control cells (Fig. 1d).

To explore cell morphology during the rolling of control and *Rap1*KD cells expressing green fluorescent protein (GFP), cells were infused into the flow chamber, and their GFP fluorescence was recorded under shear flow. As shown in Fig. 1e, *Rap1*KD cells, which rolled slowly (<100 μm s^−1^), exhibited several tether-like membrane extension^22^. When the tether adhered to the endothelium and extended upstream of the flow, its instantaneous velocity was reduced to less than 50 μm s^−1^ (Fig. 1e and Supplementary Fig. 1e). By contrast, control cells, which rolled at >400 μm s^−1^, did not extrude clear protrusions on the endothelium (Fig. 1e and Supplementary Fig. 1e). There was no difference between control and *Rap1*KD cells in the cellular localization of L-selectin, whereas *Rap1*KD cells exhibited several blebs containing L-selectin (Supplementary Fig. 2a).

Previous papers described that both tethers and blebs are expanded by uncoupling between the plasma membrane and actin cortex^23^,^24^,^25^,^26^. We examined bleb formation under a static condition in Rap1-deficient cells expressing LifeAct-mCherry, which was used as a marker of actin polymerization; membrane bleb expansion does not involve actin polymerization events, which distinguishes blebs from all other known cell protrusions. Rap1-deficient cells exhibited active membrane blebbing in the absence of chemokine stimulation (Fig. 1f and Supplementary Movies 5 and 6). As shown in Supplementary Fig. 2b, the bleb was expanding without actin polymerization, then it occurred at the bleb cortex to halt the bleb expansion and the bleb was retracted^27^. The number, size and duration of blebs were significantly higher in *Rap1*KD cells than in control cells (Fig. 1f).

Taken together, these data suggest that Rap1-GDP suppresses L-selectin-dependent rolling by limiting tether formation.

### Rap1 deficiency impaired T-cell homeostasis

**Figure 2:**
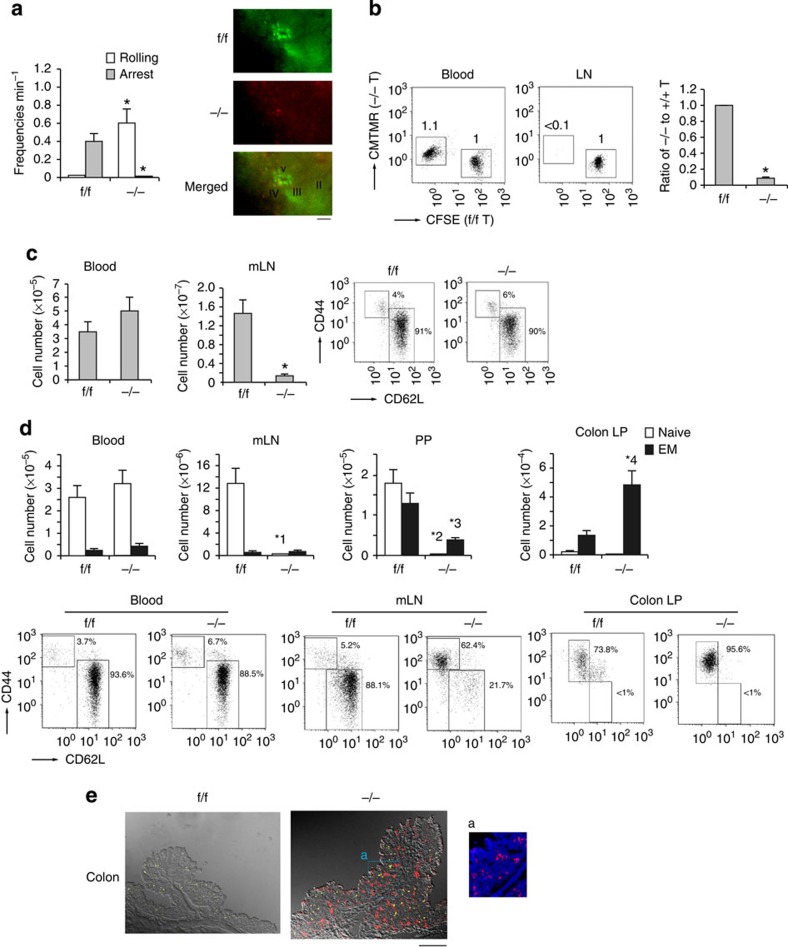
Distribution of CD4^+^ T cells in the lymphoid tissues of −/− mice. (**a**; Left) Percentages of rolling or arrest of adoptively transferred f/f or −/− T cells passing through HEVs in the mLNs. Data represent the means±s.e.m. of four independent experiments. *P<0.001 versus f/f T cells. (Right) Appearance of T-cell attachment to the HEV. Intravital images were taken 30 min after intravenous transfer of differently labelled T cells from *Rap1*^f/f^ (green) and *Rap1*^−/−^(red) mice. Scale bars, 50 μm. (**b**; Left) *Rap1a*, *b*^f/f^ and *Rap1a*, *b*^−/−^ T cells were labelled with CFSE and CMTMR, respectively, mixed in equal numbers and injected into normal mice. After 1 h, lymphocytes from the blood and mLNs of the recipient mice were analysed, and their flow-cytometry profiles are shown. Numbers besides the boxed areas indicate the ratio of −/− to f/f cells. (Right) Ratios of −/− to f/f T cells. *P<0.001 versus f/f T cells. (**c**; Left) Numbers of T cells in blood and mLNs from f/f and −/− mice at 4–5 weeks of age (*n*=10). *P<0.001 versus f/f mice. (Right) Representative flow-cytometric profiles of lymphocytes from mLNs of f/f or −/− mice. Graphs show expression of CD44 and CD62L on CD3^+^-gated lymphocytes. (**d**; Upper) Numbers of naive and effector/memory CD4^+^ T cells in the blood, mLNs, PPs and colon LP of f/f or −/− mice (*n*=10) at 9 weeks of age. *^1^*P*<0.001,*^2^*P*<0.001, *^3^*P*<0.005, *^4^*P*<0.001 versus corresponding f/f mice. (Lower) Flow-cytometric profiles of the blood, mLNs and colon LP of f/f or −/− mice. Graphs show expression of CD44 and CD62L on CD4^+^-gated lymphocytes. (**e**) Increased infiltration of CD4^+^ T cells in the colon of −/− mice. Frozen sections of the colon from f/f or −/− mice were stained with anti-CD4 (red), CD8 (yellow) and DAPI (blue). Low (× 40) and high (× 200; **a**) magnification images of colon from f/f or −/− mice are shown. Scale bars, 200 μm. All data show the means±s.e.m. or representative of three independent experiments.

The frequencies of rolling on the HEV was significantly higher in *Rap1*^−/−^ T cells than in *Rap1*^f/f^ T cells, whereas *Rap1*^−/−^ T cells did not accumulate on the HEV of mLNs 30 min after adoptive transfer of a mixture of similar numbers of differentially labelled *Rap1*^f/f^ and *Rap1*^−/−^ T cells (Fig. 2a). *Rap1*^−/−^ T cells homed to peripheral LNs less than 10% as efficiently as *Rap1*^f/f^ T cells (Fig. 2b). Consistent with these data, the number of T cells in peripheral lymph nodes of *Rap1*^−/−^ mice diminished to 10% of the level in *Rap1*^f/f^ mice at 4–5 weeks of age. Concomitantly, there was an increase in the number of T cells in the blood of *Rap1*^−/−^ mice (Fig. 2c). More than 90% of both *Rap1*^−/−^ and *Rap1*^f/f^ T cells exhibited a naive phenotype (Fig. 2c).

However, at ages more than 8 weeks, the ratios of CD62L^−^CD44^+^ T_EM_ cells in the mLNs and PPs of *Rap1*^−/−^ mice increased to 62.5+4.3 and 84.2+6.4%, whereas 5.8+1.8 and 39.3+3.2% in *Rap1*^f/f^ mice (Fig. 2d). T_EM_ cell number in the blood and mLNs did not significantly differ between *Rap1*^f/f^ and *Rap1*^−/−^ mice, and T_EM_ cell number in the PPs was lower in *Rap1*^−/−^ mice than in *Rap1*^f/f^ mice (Fig. 2d). Nevertheless, the number of T_EM_ cells in the colon LP of *Rap1*^−/−^ mice increased more than fourfold relative to *Rap1*^f/f^ mice, in contrast to the impaired homing of Rap1-deficient T_N_ cells into peripheral LNs (Fig. 2d). Immunostaining showed that CD4^+^ T cells but not CD8^+^ cells infiltrated in the colon LP of *Rap1*^−/−^ mice (Fig. 2e). The ratios of gut-tropic T_EM_ cells expressing high expression of *α*_4_*β*_7_ in the mLNs of *Rap1*^f/f^ and *Rap1*^−/−^ mice were exactly the same (Supplementary Fig. 1b). These data suggest that Rap1 deficiency accelerated T_EM_ to home into the colon.

### Rap1 CKO mice developed T-cell-dependent colitis

**Figure 3:**
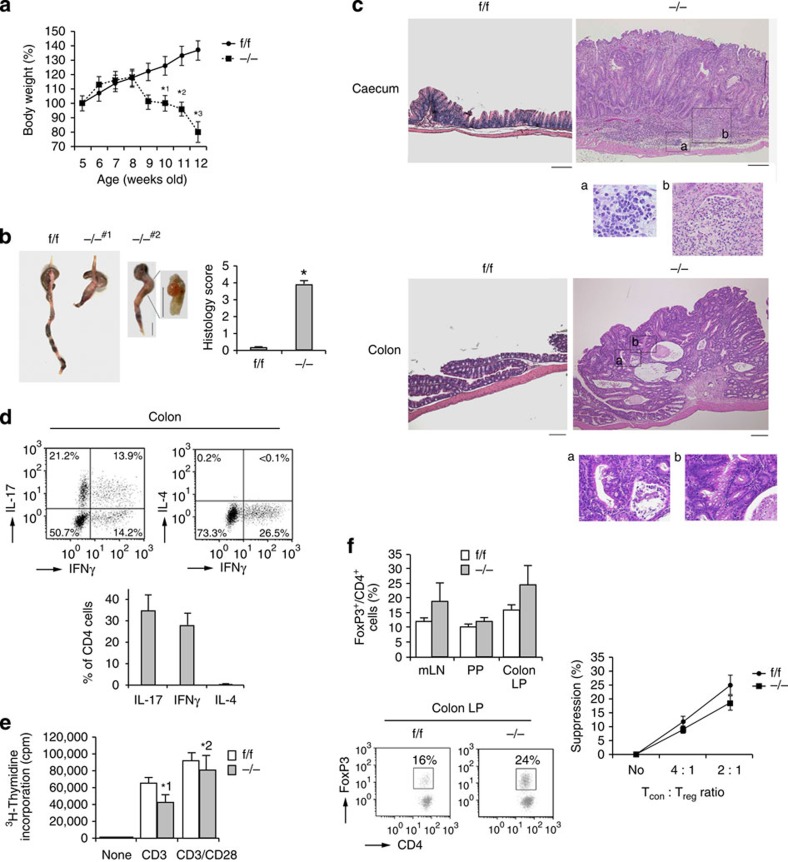
*Rap1* CKO mice develop spontaneous colitis with adenoma. (**a**) Weight loss of −/− mice. Body weights of f/f or −/− mice (*n*=10) were presented as percentage of original body weight. Data represent means±s.e.m. *^1^*P*<0.02, *^2^*P*<0.01, *^3^*P*<0.001 versus the corresponding f/f mice. (**b**; Left) Representative colon morphology of f/f or −/− mice at 8–12 weeks of age. #1 Shows intussusception in the colon. #2 Shows adenoma between caecum and colon. Scale bar, 1 cm. (Right) Light microscopic assessment of damages of colitis. Data represent means±s.e.m. (*n*=10 in each group), *P<0.001 versus f/f mice. (**c**) Histology of intestinal inflammation (upper) and adenoma (lower) in −/− mice. Representative low (× 40) and high (× 200; **a**,**b**) magnification histological images of large intestines. Upper a and b represent lymphocyte infiltration and loss of glands, respectively, and lower a and b represent mucosal dysplasia. Scale bars, 200 μm. (**d**; Upper) Representative cytokine profiles of T cells from the colon LP of −/− mice with colitis (*n*=5). T cells from the colon LP were subjected to flow cytometry to determine the frequency of T_H_1 and T_H_17, or T_H_1 and T_H_2, based on their production of IFN-γ, IL-17 and IL-4. (Lower) Graphs represent the means±s.e.m. of the percentages of IL-17-, IFNγ- or IL-4-producing CD4^+^ cells in the colon. (**e**) [^3^H]-thymidine uptake by f/f or −/− CD4^+^ T_N_ cells. Naive CD4^+^ T cells from f/f or −/− mice were unstimulated (none) or stimulated with anti-CD3, ±anti-CD28. Data represent the means±s.e.m. of three independent experiments.*^1^*P*<0.01, *^2^*P*<0.02 versus f/f T cells. (**f**; Left upper) Percentages of Foxp3^+^ cells (relative to all CD4^+^ T cells) in the mLNs, PPs and colon LP of f/f or −/− mice (*n*=10) at 9 weeks of age. Data represent means±s.e.m. (Left lower) Representative flow-cytometric profiles of the colon. LP of f/f or −/− mice. (Right) Treg-mediated suppression. Control CD4^+^ T_N_ cells (T_con_) were mixed at the indicated ratios with f/f (●) or −/− (▪) Treg cells (Treg).

*Rap1*^−/−^ mice developed wasting disease, characterized by a progressive loss of body weight and the appearance of diarrhoea, at the age of 9 weeks (Fig. 3a). At 12 weeks, intussusception and anal prolapse were observed in *Rap1*^−/−^ mice, and histological analysis of tissue revealed that all mice developed severe colitis, with filtration of lymphocytes, epithelial hyperplasia, deletion of goblet cells and loss of glands (Fig. 3b,c). The mice sometimes exhibited crypt abscesses and epithelial granuloma characteristic of Crohn's disease, but did not exhibit severe ulceration or neutrophil infiltration (Supplementary Fig. 2c), in contrast to colonic injury-dependent inflammation such as dextran sodium sulphate-induced colitis, which is independent of T cells^28^. Furthermore, these symptoms always accompanied the development of colitis-associated tubular adenomas with low-grade dysplasia (Fig. 3c). Analysis of T_EM_ cells accumulated in the colon LP of *Rap1*^−/−^ mice with colitis revealing that pathogenic T_H_17 and T_H_1 effector cells generated in mLNs and PPs homed to the colon and induced intestinal inflammation (Fig. 3d).

Next, we stimulated CD4^+^ T_N_ cells from *Rap1*^f/f^ and *Rap1*^−/−^ mice by the crosslinking with anti-CD3 and anti-CD28 antibodies, and examined the effects of Rap1 deficiency on the proliferative response and cytokine production. As shown in Fig. 3e, Rap1-deficient T cells exhibited reduced proliferation in response to crosslinking of the TCR complex in the presence or absence of anti-CD28, compared with control T cells. There was no difference between *Rap1*^f/f^ and *Rap1*^−/−^ T cells in the production of interferon gamma (IFNγ), interleukin (IL)-10, tumour necrosis factor (TNF) and IL-6, whereas production of IL-2, IL-4 and IL-17 was decreased or slightly increased in *Rap1*^−/−^ T cells relative to *Rap1*^f/f^ T cells (Supplementary Fig. 2d). The ratio and suppressive function of Foxp3^+^ T cells did not decrease in mLNs, PPs or colon LP of *Rap1*^−/−^ mice, compared with *Rap1*^f/f^ mice (Fig. 3f). These data suggest that the elevation of T_H_17 or T_H_1 effector cells in the large intestine of *Rap1*^−/−^ mice was not dependent on the augmented proliferation or cytokine production in Rap1-deficient T cells.

### Rap1 deficiency facilitated rolling of T_EM_ cells

**Figure 4:**
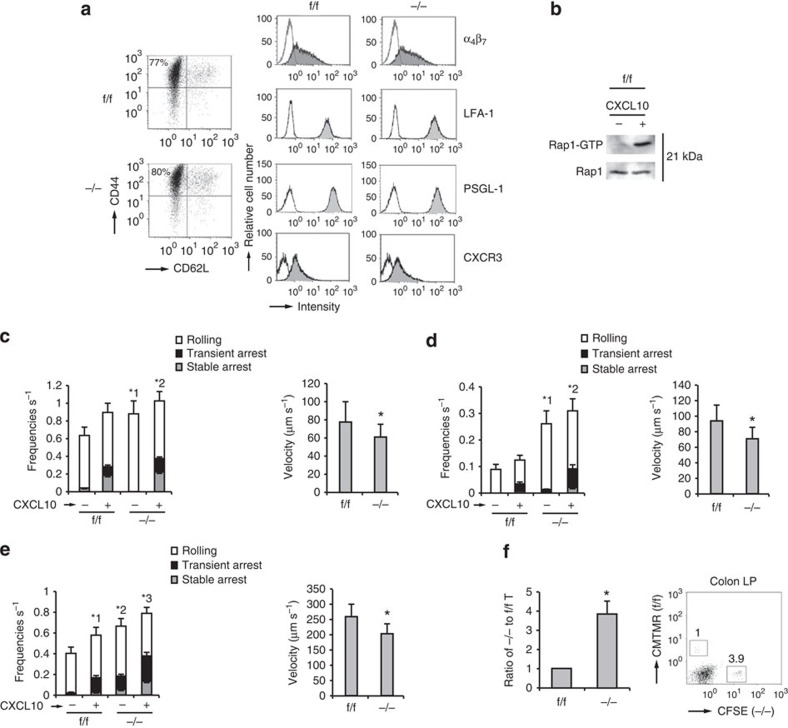
Rap1 deficiency enhances rolling of T_EM_ cells. (**a**) Representative expression profiles of CD44 and CD62L, α_4_β_7_, LFA-1, PSGL-1 and CXCR3 in CD4^+^T cells from f/f or −/− mice, cultured under T_H_17-polarizing conditions. (**b**) f/f T_EM_ cells were incubated at 37 °C at 15 s in the presence or absence of CXCL10. Cell lysates were pulled down with GST-RalGDS-RBD and immunoblotted with anti-Rap1. (**c**; Left) f/f or −/− T_EM_ cells were perfused on LS12 cells expressing P-selectin and mouse ICAM-1 ±CXCL10. Adhesion of more than 100 cells was measured. *^1^*P*<0.002, *^2^*P*<0.05 versus total frequency of the corresponding f/f T_EM_ cells. (Right) The rolling velocity of f/f or −/− T_EM_ cells without CXCL10. *P<0.03 versus f/f T_EM_ cells. (**d**; Left) f/f or −/− T_EM_ cells were perfused on LS12 cells expressing mouse MadCAM-1 ±CXCL10. Adhesion of more than 100 cells was measured. *^1^*P*<0.001, *^2^*P*<0.002 versus total frequency of the corresponding f/f T_EM_ cells. (Right) The rolling velocity of f/f or −/− T_EM_ cells without CXCL10. *P<0.01 versus f/f T_EM_ cells. (**e**; Left) f/f or −/− T_EM_ cells were perfused on plates immobilized with MadCAM-1 ±CXCL10. Adhesion of more than 100 cells was measured. *^1^*P*<0.02 versus unstimulated f/f T_EM_ cells. *^2^*P*<0.01, *^3^*P*<0.03 versus total frequency of the corresponding f/f T_EM_ cells. (Right) The rolling velocity of f/f or −/− T_EM_ cells on MadCAM-1 without CXCL10. *P<0.005 versus f/f T_EM_ cells. (**f**) Homing assay of T_EM_ cells into the colon. (Left) f/f or −/− T_EM_ cells labelled with CMTMR or CFSE were injected into normal mice. After 10 h, cells from the colon LP of recipient mice were analysed using flow cytometry. The ratios of −/− to f/f T_EM_ cells are shown (*n*=3 experiments). (Right) Flow-cytometry profile of colon LP in the recipient mice. Numbers indicate the ratio of −/− to f/f T_EM_ cells. All data show the means±s.e.m. or are representative of three independent experiments.

To compare the homing ability of Rap1-deficient T_EM_ cells, CD4^+^ T_N_ cells from *Rap1*^f/f^ and *Rap1*^−/−^ mice were cultured under T_H_17-polarizing conditions. Rap1 deficiency exerted no significant effects on differentiation into T_H_17 and T_H_1 cells or expression of α_4_β_7_, LFA-1, PSGL-1 and CXCR3 (Fig. 4a and Supplementary Fig. 3a). Rap1 in f/f T_EM_ cells was activated after CXCL10 stimulation (Fig. 4b). We investigated whether Rap1 deficiency influenced the adhesion cascades of T_EM_ cells on the endothelium expressing P-selectin and ICAM-1 under shear flow. *Rap1*^−/−^ T_EM_ cells exhibited significantly more frequent and slow rolling in the absence or presence of CXCL10, relative to *Rap1*^f/f^ T_EM_ cells (Fig. 4c). These data demonstrated that PSGL-1/P-selectin-dependent rolling was elevated in *Rap1*^−/−^ T_EM_ cells.

Next, we investigated the effects of Rap1 deficiency on the adhesion cascades of T_EM_ cells on the endothelium expressing MadCAM-1 under shear flow. *Rap1*^−/−^ T_EM_ cells showed more frequent and slow rolling, relative to *Rap1*^f/f^ T_EM_ cells in the absence or presence of CXCL10 (Fig. 4d). We also examined the rolling and arrest of Rap1-deficienct T_EM_ cells on the purified MadCAM-1 under shear flow. *Rap1*^−/−^ T_EM_ cells exhibited significantly more frequent rolling and arrest in the absence or presence of CXCL10, relative to *Rap1*^f/f^ T_EM_ cells (Fig. 4e and Supplementary Movies 7 and 8). The rolling speed of Rap1-deficient T_EM_ cells was significantly slower than that of *Rap1*^f/f^ T_EM_ cells (Fig. 4e). The expression of constitutively negative mutant Rap1N17 in *Rap1*^−/−^ T_EM_ cells significantly reduced the frequencies of adhesion events on the MadCAM-1 (Supplementary Fig. 3b). *Rap1*^−/−^ T_EM_ cells, which rolled slowly (<100 μm s^−1^), exhibited several tether-like membrane extension, similarly to L-selectin-dependent rolling, whereas rolling control cells did not extrude clear protrusions on the MadCAM-1 (Supplementary Fig. 3c). To compare the ability of *Rap1*^−/−^ and *Rap1*^f/f^ T_EM_ cells to home into the colon, we differentially labelled these cells and injected them into normal mice. Consistent with the exacerbated rolling and arrest on the MadCAM-1, the number of Rap1-deficient T_EM_ cells that homed into colon was approximately fourfold higher than in *Rap1*^f/f^ mice (Fig. 4f).

These data suggest that Rap1 deficiency accelerated the homing of pathogenic T_EM_ cells into the large intestine, which might contribute to the induction of spontaneous colitis.

### Diminished phosphorylation of ERM in Rap1-deficient cells

**Figure 5:**
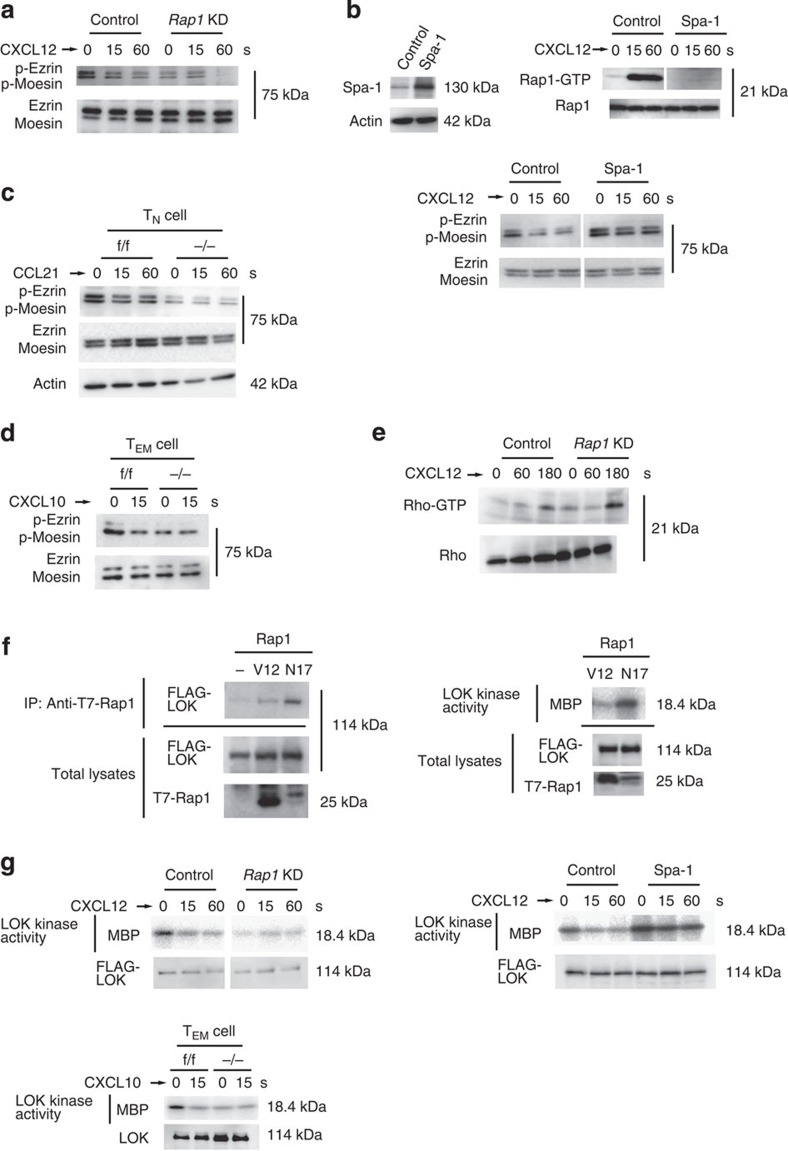
Reduced phosphorylation of ERM in *Rap1*-deficient cells. (**a**) Control or *Rap1*KD cells stimulated with CXCL12 as indicated. Cell lysates were probed with antibody against ERM phosphorylated at Thr 567 and 558, or anti-ERM. (**b**; Left) Lysates from BAF cells transfected with vector (control) or Spa-1 immunoblotted with anti-Spa-1. Actin is a loading control. (Right upper) Cells stimulated with CXCL12 as indicated. Cell lysates were pulled down with GST-RalRBD, and immunoblotted with anti-Rap1. (Right lower) Control or Spa-1-expressing cells were stimulated with CXCL12 as indicated. Cell lysates were probed with anti-phosphorylated ERM, or anti-ERM. (**c**) Phosphorylation of ERM in f/f or −/− CD4^+^ T_N_ cells. T_N_ cells were stimulated with CCL21 as indicated. Total lysates from f/f or −/− T_N_ cells were probed with antibody against phosphorylated ERM or anti-ERM. Actin is a loading control. (**d**) Phosphorylation of ERM in f/f or −/− CD4^+^ T_EM_ cells. T_EM_ cells were stimulated with CXCL10 for 15 s. Total lysates from f/f or −/− T cells were probed with antibody against phosphorylated ERM, or anti-ERM. (**e**) Control or *Rap1*KD cells were stimulated with CXCL12 as indicated. Cell lysates were pulled down with GST-Rhotekin RBD and immunoblotted with an anti-Rho antibody. (**f**) COS cells were transfected with FLAG-LOK, together with T7-tagged Rap1V12 or Rap1N17. (Left) Cell lysates were immunoprecipitated with anti-T7 and immunoblotted with an anti-FLAG antibody. (Right) Kinase activity of LOK in Rap1N17 or V12-expressing COS cells measured using Myelin basic protein (MBP) as the substrate. Total FLAG-LOK or T7-Rap1 was immunoblotted with an anti-FLAG or T7 antibody. (**g**; Left upper) Control or *Rap1*KD cells transfected with FLAG-LOK were stimulated with CXCL12 as indicated. Kinase activity of LOK was measured. Total FLAG-LOK was immunoblotted with an anti-FLAG antibody. (Left lower) Kinase activity of LOK in f/f or −/− T_EM_ cells stimulated with CXCL10 for 15 s. Total LOK was immunoblotted with an anti-LOK antibody. (Right) Kinase activity of LOK in control or Spa-1-expressing cells stimulated with CXCL12 as indicated. Total FLAG-LOK was immunoblotted with an anti-FLAG antibody. For all blots, a representative of three or four independent experiments is shown.

We investigated whether the ERM proteins were regulated by Rap1-GDP because they play a critical role in bleb formation^26^,^29^,^30^. As shown in Fig. 5a, ezrin and moesin were mainly expressed in lymphoid cells. These proteins were highly phosphorylated at thr-567 and -558 in unstimulated control BAF cells; however, the phosphorylation level was reduced to less than half after CXCL12 stimulation (Fig. 5a). On the other hand, basal phosphorylation of ERM in unstimulated *Rap1*KD cells was 38.5+6.5% of that in control cells (Fig. 5a), suggesting that Rap1-GDP is necessary for their maximal phosphorylation in resting cells. CXCL12-induced uropod formation, which is dependent on phosphorylation of ERM^31^, was also impaired in *Rap1*KD cells (Supplementary Fig. 4a). The expression of Spa-1 inhibited the activation of Rap1 by CXCL12 stimulation, which peaked at 15 s after stimulation (Fig. 5b). The phosphorylation of ERM was approximately 1.6-fold higher than in control cells at all points after CXCL12 stimulation (Fig. 5b), suggesting that Rap1 activation is involved in reduction of ERM phosphorylation.

ERM proteins were predominantly present in their active conformations in resting T_N_ cells through their phosphorylation at Thr-567 and -558, which declined at 15 s after CCL21 stimulation^17^,^32^. The basal level of ERM phosphorylation in *Rap1*^−/−^ T_N_ cells was reduced to 47.5+12.7% of that in resting *Rap*1^f/f^ T_N_ cells (Fig. 5c). Importantly, ERM proteins in resting T_EM_ cells were also highly phosphorylated; however, the phosphorylation level decreased quickly on CXCL10 stimulation (Fig. 5d). In the absence of CXCL10, phosphorylation levels of ERM proteins in Rap1-deficient T_EM_ cells was 36.3+5.4% of that in f/f T_EM_ cells (Fig. 5d).

Since Rho-associated kinase (Rock) phosphorylates ERM proteins^33^, we investigated whether Rho activity was affected by the Rap1 deficiency. Rho was activated 3 min after CXCL12 stimulation in control cells; however, there was no reduction in the *Rap1*KD cells (Fig. 5e). Next, we examined the relationship between LOK and Rap1 because LOK phosphorylates ERM in resting lymphocytes^19^. Rap1-GDP but not Rap1-GTP associated with LOK, and the association of LOK with Rap1-GDP increased the kinase activity of LOK (Fig. 5f). Consistent with those data, the kinase activity of LOK in *Rap1* KD cells was reduced to 18+2.6% of that of control cells (Fig. 5g). CXCL12 stimulation rapidly reduced the kinase activity of LOK to one-third, which was consistent with the kinetics of reduction of the phosphorylation of ERM proteins. The kinase activity of LOK in Rap1-deficient T_EM_ cells was also reduced to 38.5+8.5% of that of control cells without CXCL10 stimulation (Fig. 5g). In Spa-1-expressing cells, the basal kinase activity increased to 1.8 times and hardly decreased after CXCL12 stimulation, indicating that the conversion to Rap1-GTP is important for reduction of LOK kinase activity by chemokine (Fig. 5g). These data suggest that Rap1-GDP is required for LOK activation and plays an important role in the phosphorylation of ERM proteins in resting cells.

### Phosphomimetic ezrin or active LOK reduced rolling

**Figure 6:**
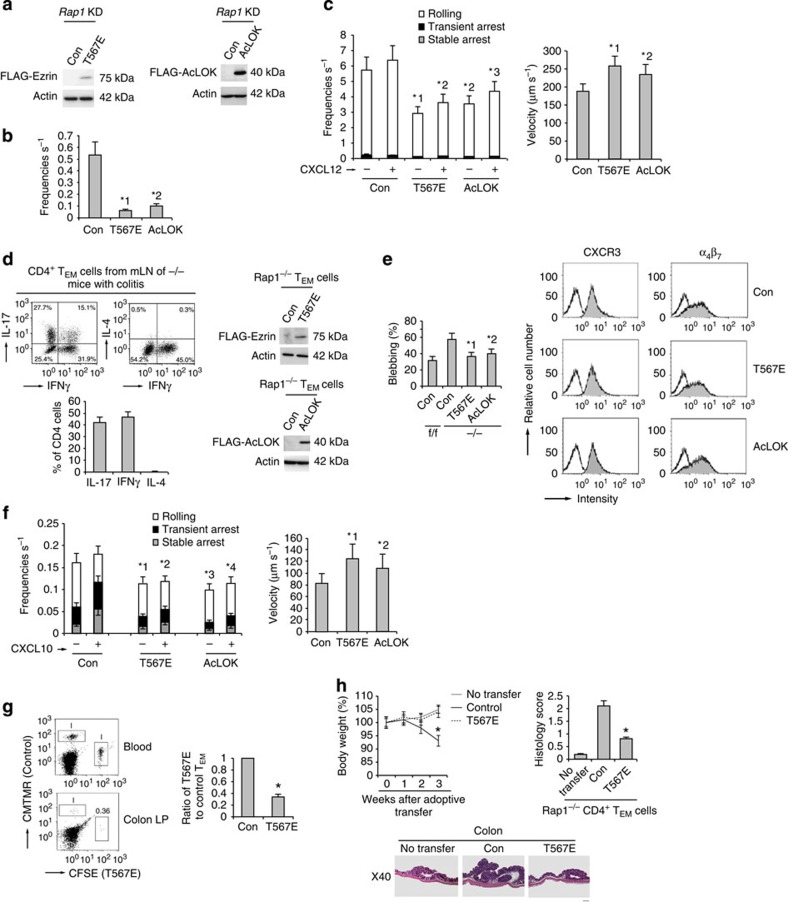
Phosphomimetic ezrin or AcLOK inhibits T-cell rolling. (**a**) Western blots with anti-FLAG of total cell lysates from *Rap1*KD cells with control, FLAG-Ezrin mutant (T567E; left) or FLAG-AcLOK (right)-encoding lentivirus. Actin is a loading control. (**b**) Adhesion of control, T567E or AcLOK-expressing *Rap1*KD cells to purified CD300LG under shear flow. *^1^*P*<0.001, *^2^*P*<0.001 versus control *Rap1*KD cells. (**c**; Left) Adhesion of control, T567E or AcLOK-expressing *Rap1*KD cells to the endothelium under shear flow. *^1^*P*<0.001, *^2^*P*<0.003, *^3^*P*<0.002 versus frequencies of the corresponding control *Rap1*KD cells. (Right) Rolling velocities without CXCL12 (*n*=150). *^1^*P*<0.01, *^2^*P*<0.02 versus control *Rap1*KD cells. (**d**; Left upper) Cytokine profiles of CD4^+^ T_EM_ cells from mLNs of −/− mice with colitis (*n*=5). (Left lower) Percentages of IL-17, IFNγ or IL-4-producing cells. (Right) Western blots with anti-FLAG of total cell lysates from −/− CD4^+^ T_EM_ cells introduced with T567E or AcLOK. Actin is a loading control. (**e**; Left) Percentages of f/f or control, T567E or AcLOK-expressing −/− T_EM_ cells blebbing more than once per minute (*n*=50). *^1^*P*<0.001, *^2^*P*<0.002 versus control −/− T_EM_ cells. (Right) Expression profiles of CXCR3 and α_4_β_7_ in those cells. (**f**; Left) Control, T567E or AcLOK-expressing −/− T_EM_ cells perfused on the MadCAM-1 ± CXCL10. Over 100 cells were measured. *^1^*P*<0.005, *^2^*P*<0.01, *^3^*P*<0.004, *^4^*P*<0.01 versus frequencies of the corresponding control −/− T_EM_ cells. (Right) Their rolling velocity without CXCL10. *^1^*P*<0.005, *^2^*P*<0.01 versus control T_EM_ cells. (**g**; Left) Analysis of cells from the colon LP of mice that received control or T567E-expressing −/− T_EM_ cells. Numbers indicate the ratios of T567E-expressing to control cells. (Right) Ratios of T567E-expressing to control cells are shown (*n*=3 experiments). *P<0.001 versus control −/− T_EM_ cells. (**h**; Upper left) Body weight of the recipient mice receiving control or T567E-expressing −/− CD4^+^ T_EM_ cells (*n*=3 per group) were presented as percentage of original body weight. *P<0.02 versus untransferred mice. (Upper right) Colitis-induced damage in the recipient mice. *P<0.001 versus control −/− T_EM_ cells. (Lower) Colon histology in recipient mice. Scale bars, 200 μm. All data show the means±s.e.m. or representative of three independent experiments.

To determine whether reduced phosphorylation of ezrin and moesin by LOK in *Rap1*KD cells enhanced L-selectin-dependent rolling, we introduced a phosphomimic mutant of ezrin (T567E) or active LOK (AcLOK) into *Rap1*KD cells (Fig. 6a). Expression of T567E ezrin or AcLOK significantly decreased the frequency of rolling of *Rap1*KD cells on purified CD300LG (Fig. 6b) or endothelium in the absence or presence of chemokine, relative to that of control cells (Fig. 6c and Supplementary Movies 9 and 10). Rolling velocity in *Rap1*KD cells was significantly increased by expression of T567E ezrin or AcLOK (Fig. 6c). In control cells, the expression of T567E ezrin also decreased the frequency of rolling on the endothelium in the presence or absence of chemokine (Supplementary Fig. 4b).

Next, we sought to determine whether T567E ezrin or AcLOK affected the adhesion cascade on MadCAM-1 under shear flow in Rap1-deficient T_EM_ cells derived from LNs of mice developing colitis, including T_H_17 and T_H_1 cells (Fig. 6d). We introduced them by lentiviral transduction while culturing the cells under T_H_17-polarizing conditions (Fig. 6d). Control, T567E ezrin or AcLOK-lentivirus-infected Rap1^−/−^ T_EM_ cells expressed similar levels of CXCR3 and α_4_β_7_, whereas blebbing in T567E ezrin or AcLOK-expressing Rap1^−/−^ T cells was significantly diminished relative to control cells (Fig. 6e and Supplementary Movies 11 and 12). As shown in Fig. 6f, the expression of T567E ezrin or AcLOK significantly suppressed the rolling and subsequent arrest of Rap1^−/−^ T_EM_ cells in the absence or presence of CXCL10, and also increased their rolling velocity.

Consistent with that observation, the ability of T567E ezrin-expressing Rap1^−/−^ cells to home into the colon was reduced by 36.5+5.3% relative to control cells, although they were equally present in the blood (Fig. 6g). Furthermore, we adoptively transferred Rap1-deficient CD4^+^ pathogenic T cells into the radiated normal mice, and investigated whether the expression of T567E ezrin reduced the development of colitis. The recipient mice transferred with Rap1-deficient CD4^+^ pathogenic T cells developed wasting disease, characterized by a progressive loss of body weight and the appearance of diarrhoea, whereas the mice transferred with T567E ezrin-expressing cells did not after 3 weeks (Fig. 6h).

Together, these data suggest that Rap1-GDP restrains rolling behaviours via phosphorylation of ERM proteins, which inhibited the onset of colitis by suppressing the trafficking of CD4^+^-pathogenic T cells into the colon.

### FLNs suppressed LFA-1 activation

**Figure 7:**
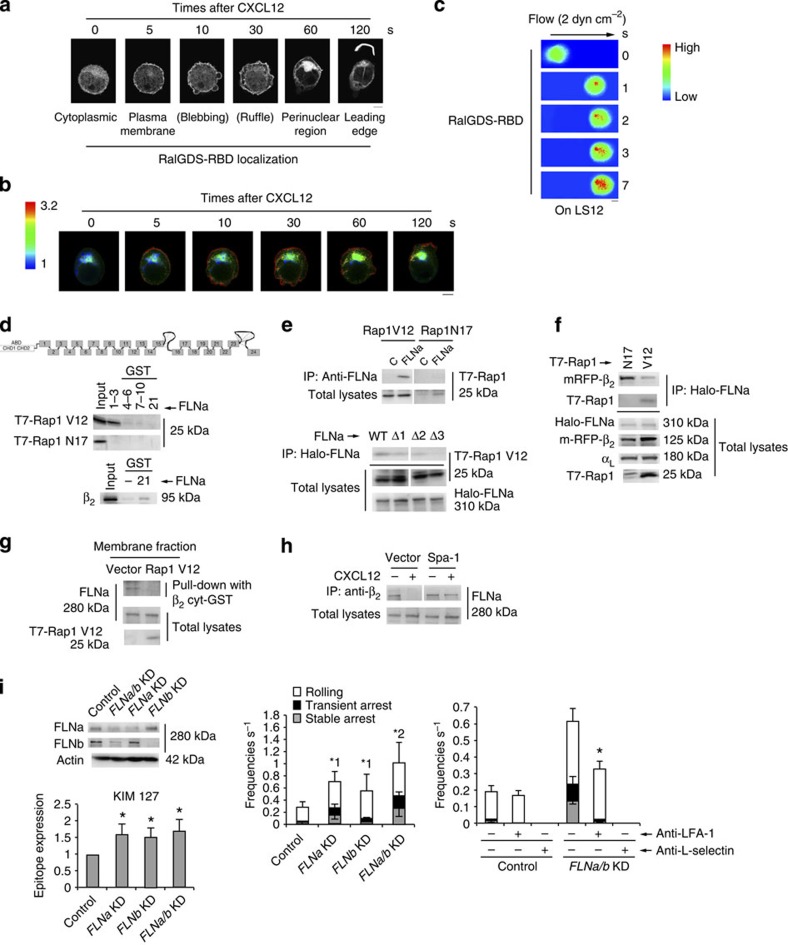
Spatiotemporal activation of Rap1 reduces association of FLN with β_2_. (**a**) RalGDS-RBD mCherry in control BAF cells stimulated with CXCL12 as indicated. Scale bar, 5 μm. (**b**) FRET-based Rap1 activity sensor-expressing control BAF cells stimulated with CXCL12 as indicated. A ratio image of mTurquoise/CFP represents FRET efficiency. Scale bar, 5 μm. (**c**) Control cells expressing RalGDS-RBD mCherry perfused on the endothelium with CXCL12. The digital images of contact areas of the cells taken under shear flow. Scale bar, 5 μm. (**d**) COS cells transfected with T7-Rap1V12, Rap1N17 or αL and β_2_ were lysed and pulled down using GST fusions of FLNa repeats 1–3, 4–6, 7–10 or 21, and immunoblotted with anti-T7 or anti-β_2_. (**e**; Upper) Lysates from COS cells transfected with T7-Rap1V12 or Rap1N17 immunoprecipitated with anti-FLNa, and immunoblotted with anti-T7. (Lower) Lysates from Cos cells transfected with Halo-FLNa wild-type and the mutants deleting repeat 1(Δ1), 2 (Δ2) or 3(Δ3), and T7-Rap1V12, precipitated using HaloLink Resin, and immunoblotted with anti-T7 or Halo. (**f**) Lysates from COS cells transfected with Halo-FLNa, T7-Rap1V12 or N17, and αL and mRFP-β precipitated with HaloLink resin, and immunoblotted with anti-mRFP, T7, Halo and αL. (**g**) Membrane fraction from BAF/LFA-1 cells transfected with vector or T7-Rap1V12 pulled down using the cytoplasmic region of β_2_-GST fusion protein. Bound FLNa and total FLNa and T7-Rap1V12 were analysed using anti-FLNa or anti-T7 antibody. (**h**) Lysates from BAF/LFA-1 cells transfected with vector or *Spa-1*, ±CXCL12 for 1 min, immunoprecipitated with anti-β_2_, and immunoblotted with anti-FLNa. (**i**; Left upper) Western blots of total cell lysates from BAF/LFA-1/L-selectin cells transduced with scramble (Control) or *FLNa* or *b*-targeting shRNA. Actin is a loading control. (Left lower) Epitope expression of monoclonal antibody KIM127. Data are normalized against LFA-1 expression detected by TS1/18. *P<0.01 versus control. (Centre) Control or *FLNa/b* KD cells perfused on LS12 monolayers (*n*=150). *^1^*P*<0.02, *^2^*P*<0.01 versus frequency of control cells. (Right) Effects of anti-L-selectin or anti-LFA-1 antibody on the adhesion events (*n*=150 per each group). *P<0.01 versus frequencies of untreated *FLNa/b* KD cells. Data represent the means±s.e.m. of triplicate experiments.

To understand the temporal and spatial regulation of Rap1 activity, we examined the dynamic subcellular localizations of Rap1-GTP after chemokine stimulation, using Ral guanine nucleotide dissociation inhibitor (GDS)-RBD (Ras-binding domain)-mCherry (RalGDS-RBD) as a reporter. RalGDS-RBD protein distributed diffusely over the cell before CXCL12 stimulation, and accumulated at the plasma membrane at 5 s after stimulation (Fig. 7a and Supplementary Fig. 5a). Blebs appeared a few seconds after Rap1 activation at the plasma membrane, followed by membrane ruffles (Fig. 7a). Several minutes after stimulation, RalGDS-RBD moved to the perinuclear region, and finally accumulated at the leading edge of polarized cells (Fig. 7a and Supplementary Fig. 5b). We also confirmed the process of Rap1 activation by Förster resonance energy transfer (FRET) using the improved Rap1 activity sensor. As shown in Fig. 7b, Rap1 activation occurred at the plasma membrane within 5 s of CXCL12 stimulation, and subsequently at the perinuclear region; ultimately, it was confined to the leading edge. Furthermore, using RalGDS-RBD, we examined the relationship between Rap1 activation and lymphocyte arrest under shear flow. When RalGDS-RBD clearly accumulated at a part of contact site between control cell and the endothelium, the cell stopped through that Rap1 activation site (Fig. 7c), suggesting that Rap1 activation at the plasma membrane within seconds by chemokine induces LFA-1-dependent arrest on the endothelium.

To investigate whether FLNs associated with the LFA-1 cytoplasmic domain as a restraint of active conformation, we examined the association of Rap1V12 or Rap1N17 with FLNa by pull-down assay using repeats 1–3, 4–6, 7–10 and 21 of FLNa-GST fusion proteins. Rap1V12, but not Rap1N17, bound to the GST (glutathione *S*-transferase)-fusion protein corresponding to repeats 1–3 best (Fig. 7d). As previously reported^34^, β_2_ of LFA-1 was associated with repeat 21 of FLNa (Fig. 7d). Rap1-GTP, but not Rap1-GDP, associated with endogenous FLNa in cells (Fig. 7e). FLNa lacking repeat 3 (Δ3) did not interact with Rap1V12 (Fig. 7e).

We next examined whether the interaction of FLNa with Rap1-GTP affects its association with the cytoplasmic region of β_2_. To this end, we transfected cells with Rap1V12 or Rap1N17 in combination with α_L_, β_2_ and Halo-tagged FLNa. FLNa was immunoprecipitated from the cell lysates using HaloLink resin and was examined for co-precipitation of β_2_ or Rap1. FLNa bound to Rap1V12, resulting in reduction of the association of FLN with β_2_ relative to that in cells expressing Rap1N17 (Fig. 7f). Endogenous FLNa associated with the β_2_ cytoplasmic region in the membrane fraction of Rap1V12-expressing cell was reduced to 32+5.4% of that of control cells (Fig. 7g). Finally, we tested whether CXCL12 stimulation decreased the interaction between FLN and β_2_ in a Rap1-GTP-dependent manner, using control or Spa-1-expressing cells. CXCL12 stimulation diminished the connection between β_2_ and FLNa in control cells (25.2+11.4%), but not in Spa-1-expressing cells (64.7+4.5%; Fig. 7h).

To clarify the roles of Rap-dependent dissociation of FLN from β_2_ in the adhesion cascade, we knocked it down more than 85% by lentiviral transduction of short hairpin RNAs specific for *FLNa* and *FLNb* (Fig. 7i). The expression levels of the activation reporter epitopes recognized by the antibodies, KIM127 and MEM148, were significantly upregulated by the knockdown of FLN (Fig. 7i and Supplementary Fig. 6a), indicating that FLN restrains the active conformation of LFA-1. The depletion of FLNa or -b in BAF/LFA-1/L-selectin increased the frequencies of rolling and arrest, and double knockdown of FLNa and -b additively increased these frequencies (Fig. 7i). The rolling velocities of *FLNa/b* KD cells were significantly lower than those of control cells (Supplementary Fig. 6b). The elevated frequencies of the interaction events in *FLNa/b* KD cells were significantly reduced by treatment with anti–LFA-1 monoclonal antibody (Fig. 7i). Rolling velocity of *FLNa/b* knockdown cells significantly increased after treatment with anti-LFA-1 (Supplementary Fig. 6c). The ablation of FLN mainly induced LFA-1-dependent slow rolling and transient arrest (Supplementary Fig. 6d).

### FLNa-deficient T cells exhibit increased LFA-1 functions

**Figure 8:**
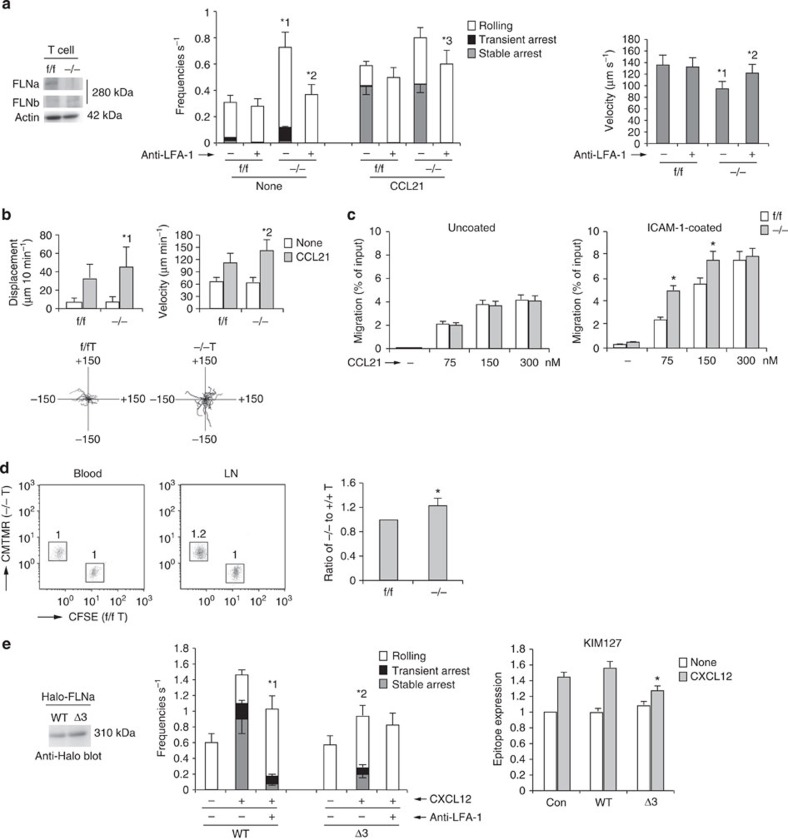
Filamin deficiency facilitates trafficking of lymphocytes. (**a**; Left) Western blotting of lysates from f/f or −/− T cells with anti-FLNa or -b. Actin is a loading control. (Centre) The adhesive events of f/f and −/− T cells with the endothelium under shear flow. *^1^*P*<0.001 versus total frequency of f/f T cells, *^2^*P*<0.001 versus total frequency of unstimulated −/− T cells,*^3^*P*<0.03 versus total frequency of stimulated −/− T cells. (Right) Effects of anti-LFA-1 on rolling velocities without CCL21. *^1^*P*<0.01 versus f/f cells. *^2^*P*<0.01, compared with untreated −/− T cells. (**b**; Upper) Displacement (left) and velocity (right) of f/f or −/− T cells on ICAM-1 in the presence or absence of CCL21 (*n*=50). *^1^*P*<0.02, *^2^*P*<0.05 versus f/f cells. (Lower) Each line represents a single-cell track of f/f or −/− T cells on ICAM-1 in the presence of CCL21. (**c**; Left) f/f or −/− T cells transmigrated through uncoated transwell in the presence of CCL21. (Right) f/f or −/− T cells transmigrated through ICAM-1-coated transwell is *P<0.002 versus corresponding f/f T cells. (**d**) f/f or −/− T cells labelled with CFSE or CMTMR were injected into normal mice. Flow cytometric profiles of blood and lymph nodes of the recipient after 1 h are shown. Numbers indicate the ratio of −/− to f/f cells. (Right) Ratios of −/− to f/f T cells are shown (*n*=4). *P<0.02 versus f/f T cells. (**e**; Left) Lysates from BAF/LFA-1/L-selectin cells introduced with Halo-tagged wild-type (WT) or Δ3 FLNa were immunoblotted with anti-Halo. (Centre) Adhesive events of those cells with the endothelium under shear flow. *^1^*P*<0.03 versus total frequency of untreated WT cells, *^2^*P*<0.01, versus total frequency of the corresponding WT cells. (Right) KIM127 expression in those cells ±CXCL12 (*n*=5 of each group). *P< 0.05 versus WT FLNa-expressing cells. All data show the means±s.e.m. or representative of three independent experiments.

We next examined the roles of FLNa in the adhesion cascade of primary T cells. To generate FLNa conditional-knockout mice, mice carrying floxed FLN alleles (*FLNa*^f/f^) were mated with lck-Cre transgenic mice to delete FLNa in T cells. Western blot analysis confirmed that the FLNa protein was not expressed in T cells derived from those mice (Fig. 8a). *FLNa*^−/−^ T cells exhibited a significant elevation in frequency, and a reduction in velocity, of LFA-1-dependent rolling, relative to *FLNa*^f/f^ T cells (Fig. 8a), but not of α_4_β_7_-dependent rolling on the MadCAM-1 (Supplementary Fig. 6e). Anti-LFA-1 treatment significantly reduced the frequencies of the interaction events and increased the rolling velocity of *FLNa*^−/−^ T cells (Fig. 8a). Displacement and migration velocity on intercellular adhesion molecule-1 (ICAM-1) were also significantly elevated in FLNa-deficient T cells relative to those in *FLNa*^f/f^ T cells (Fig. 8b). In addition, we analysed the chemotaxis of FLNa-deficient T cells towards CCL21. The number of *FLNa*^−/−^ cells that transmigrated through an ICAM-1-coated transwell filter, but not an uncoated transwell filter, was significantly elevated at low concentrations of CCL21, relative to that in *FLNa*^f/f^ cells (Fig. 8c). Homing into lymph nodes was significantly higher in FLNa-deficient T cells than in *FLNa*^f/f^ cells (Fig. 8d). These data support the idea that FLNa is a suppressor of LFA-1-dependent adhesion and migration in primary T cells.

Finally, to determine whether Rap1-dependent dissociation of FLN from β_2_ was implicated in chemokine-induced arrest, we introduced wild type and Δ3 FLNa into cells. Expression of Δ3 FLNa in BAF/LFA-1/L-selectin significantly inhibited the frequency of arrest following CXCL12 stimulation, and the extended conformation of LFA-1 recognized by the KIM127 antibody, relative to that in wild-type FLNa-expressing cells (Fig. 8e). These data indicate that Rap1-GTP binding to FLNs is important for the LFA-1 activation, which is prerequisite for arrest by chemokine stimulation.

## Discussion

In this study, we showed that Rap1-GDP restrained the tether formation and rolling behaviours of T cells through the phosphorylation of ERM via LOK (Supplementary Fig. 7). Specially, regulation of rolling of T_EM_ cells on the MadCAM-1 by Rap1-GDP restricted their homing to the colon, which contributed to prevention of T-cell-dependent intestinal inflammation. Regulation of integrin by Rap1-GTP is indispensable for the homing of T_N_ into secondary lymph nodes, but dispensable for the homing T_EM_ into the colon (Supplementary Fig. 7).

Under high shear stress due to blood flow, lymphocytes must immediately stop at the proper sites on the endothelium. Lymphocyte adhesion cascade on the HEV is achieved through multiple adhesion steps, and integrin-dependent lymphocyte arrest requires a preceding L-selectin-dependent interaction. Chemokine-dependent Rap1 activation in T_N_ cells simultaneously facilitated L-selectin- and LFA-1-dependent interaction with the endothelium. In fact, lymphocytes often stop on the endothelium without rolling, in the presence of chemokine. Simultaneous induction of L-selectin- and integrin-mediated adhesion by Rap1-GTP provides an effective ‘brake' for circulating lymphocytes in the blood, and is crucial for preventing lymphopenia in peripheral lymph nodes. Our previous reports demonstrated that the expression of dominant active form of Rap1, Rap1V12, enhanced markedly LFA-1-ICAM-1 adhesion under static conditions, whereas it did not dramatically increase the frequencies of stable arrest under shear flow. These data indicate that Rap1 is indispensable but not enough for firm adhesion under shear flow, which might require other signals generated via G protein coupled receptor (GPCR) such as other Rho family GTPases.

Defective LFA-1 adhesion in Rap1 CKO mice resulted in T-cell lymphopenia: lymphocyte numbers were reduced to less than 10% of the control values in peripheral LNs. Homeostatic proliferation in peripheral lymphoid organs is elicited by lymphopenia and contributes not only to the maintenance of T cell but also to the generation of pathogenic T cells causing inflammatory diseases, including inflammatory bowel diseases^35^. Severe lymphopenia in mLNs of Rap1-deficient CKO mice might promote generation of autoreactive T_EM_ cells as a result of homeostatic proliferation. However, we cannot exclude the possibility that Rap1 dependency affects the T-helper-cell fate decisions.

It takes several minutes after chemokine stimulation to induce stable adhesion and polarization in lymphocytes; these phenomena are mediated by RAPL–Mst1, downstream effector molecules of Rap1 (ref. 36; Supplementary Fig.7). On the other hand, LFA-1 activation by Rap1-GTP occurs within seconds after chemokine stimulation. Activation of Rap1 at the perinuclear region subsequent at the leading edge might be implicated in Mst1–Rab13-dependent delivery of LFA-1 (ref. 37), and Rap1-GTP on the plasma membrane might release FLNs from LFA-1 (Supplementary Fig.7). These results suggest that Rap1-GTP plays distinct roles at different cellular compartments. Thus, Rap1 activation might be spatially and temporally activated by different GEFs. To understand the regulation of T-cell homeostasis, it is important to identify Rap1 GEFs involved in chemokine-mediated signalling and clarify how Rap1 activation is regulated by them.

LOK, a member of the STE20-like kinase family, is expressed predominantly in lymphoid organs^38^. LOK is abundant at the plasma membrane, and phosphorylates ERM proteins and prevents lymphocyte polarization and migration in resting lymphocytes^19^. Active ERM proteins inhibit bleb growth, thereby forward movement in cell migration and endothelial transmigration^39^. Consistent with those data, our study showed that the kinase activity of LOK was quickly downregulated by chemokine stimulation, as well as reduction in the phosphorylation of ERM proteins. Therefore, Rap1-GDP-LOK might control not only the adhesion cascade on MadCAM-1, but also endothelial transmigration into the colon.

FLN and talin directly compete for integrin cytoplasmic tail binding, suggesting that FLN might exert its inhibitory effect by displacing talin from integrin cytoplasmic tails^40^. After the release of FLN from β_2_, the head domain of talin is believed to associate with the cytoplasmic domain of β_2_ and trigger a separation of α_L_ and β_2_, resulting in a transition from a bent to an extended conformation. FLN binds integrin β_1_ cytoplasmic tails and inhibits integrin-dependent cell migration. Knockdown of FLN in NIH3T3 cells promotes the activation of β_1_ integrins^41^. This study indicates that FLN suppresses β_2_ integrin function in lymphocytes.

Extensive colitis in mice with Rap1-deficient T cells was associated with the tubular adenomas at 12 weeks of age. In human, ulcerative colitis is characterized by repeated inflammation that can lead to oncogenic insults to the colonic epithelial^42^. In these mice, tumours appeared at short period after the development of colitis. Therefore, these mice provide a useful spontaneous model for investigating the pathogenesis of colitis-associated tumours.

## Methods

### Cell culture and mice

BAF mouse pro-B cell lines, which were derived from the bone marrow of Balb/c mice, were maintained with RPMI 1640 medium (GibcoBRL) containing 10% fetal calf serum (FCS), 50 μm β-mercaptoethanol and 5% WEHI-3 conditioned medium as a source of interleukin 3 (ref. 43). CD3^+^ or CD4^+^ T cells were isolated from the lymph nodes or spleen of control, Rap1- or FLN-deficient mice by MidiMACS (Miltenyi Biotec) or sorting on a moFloXDP (Beckman Coulter). *Rap*1a^f/f^ mice containing floxed exons 2–3 of *Rap1a*, *Rap1b*^f/f^ mice containing floxed exon 1 of *Rap1b* and *Flna*^f/f^ mice containing floxed exons 2–7 of *Flna* (The Jackson Laboratory) were crossed with wild-type C57BL/6 mice and maintained under specific pathogen-free conditions. Those mice were crossed with lck-Cre or CD4-Cre mice, yielding mice with T-cell-specific deletion of *Rap1a/b* or *Flna*. In all experiments, 4–12-week-old littermates (both male and female) were used. All experiments were in accordance with protocols approved by the Animal Care and Use Committee of Kitasato University (Kanagawa, Japan).

### Antibodies and reagents

Fluorescence-conjugated anti-mouse CD3, CD4, CD8, CD62L, CD44, CXCR3, CCR7, α4β7, Foxp3 (eBioscience ), anti-mouse LFA-1 (Santa Cruz Biotechnology), anti-human LFA-1 (TS2/4, TS1/18; American Type Culture Collection), anti-T7 (Novagen), anti-RFP (MBL), anti-Spa1, anti-LOK, anti-FLNa, anti-FLNb (Bethyl), anti-Rap1 (BD Biosciences), anti-ERM, anti-pERM (Cell Signaling), anti-activation epitope of LFA-1 (MEM148; Abcam), anti-FLAG, β-actin (Sigma) and anti-Halo, HaloLink resin (Promega), and peroxidase-conjugated goat anti-rat or -mouse IgG (Cell Signaling) were used for flow cytometry (1:100), immunoprecipitation (1:500) and immunoblotting (1:1,000). Anti-activation epitope of LFA-1(KIM127) was kindly donated by Dr T. Springer (Harvard Medical School, Boston). Mouse CXCL12, CCL21 and CXCL10 were purchased from R&D Systems. Cytokine production was measured using BD Cytometric Bead Array (CBA) solutions (BD Biosciences).

### DNA constructs and transfection

A cDNA sequence encoding repeats 1–3, 4–6, 7–10 and 21 of FLNa, or the cytoplasmic region of β_2_, was subcloned into the pGEX vector (GE Healthcare Bio-Sciences) and expressed in *Escherichia coli* BL21 as a GST-fusion protein. Halo-tagged *FLNa*, MadCAM-1 and P-selectin cDNA were purchased from Kazusa DNA Research Institute and subcloned into pcDNA3.1 (Life Technologies) or a lentivirus vector (CSII-EF-MCS; a gift from H. Miyoshi, RIKEN, Wako, Japan). We deleted repeat 3 of *FLNa* using the KOD-Plus-mutagenesis kit (TOYOBO), and subcloned it into pcDNA3.1. Human α_L_ and β_2_ subunits of LFA-1 were kindly donated by Dr T. Springer (Harvard Medical School). LifeAct was fused to mCherry^37^. Phosphomimetic human ezrin was produced from cDNA (Kazusa DNA Research Institute) by a single point replacement of threonine with glutamic acid at position 567 using the mutagenesis kit and subcloned mutant ezrin N-terminally tagged with the FLAG epitope into the NotI site of pcDNA3.1. To produce a mammalian expression vector of enhanced GFP (EGFP)-tagged Ral-GDS-RBD, we subcloned cDNAs encoding EGFP and the RBD of human Ral-GDS (amino acids 772–868) into the NheI/HindIII and BamHI/EcoRI sites of pcDNA3.1, respectively. An improved FRET-based Rap1 activity sensor, consisting of an improved version of yellow fluorescent protein, Venus-tagged Rap1 linked with a monomeric Turquoise (mTurquoise)-tagged RAPL-RBD, was constructed in pcDNA3.1. Constitutively active (Rap1V12) or negative (Rap1N17, A17) mutants of human Rap1a were produced from their cDNAs by a single point replacement of glycine with valine at position 12, or of serine with asparagine or alanine at position 17 (refs 3, 20, 43), subcloned into pcDNA3.1 or a lentivirus vector (CSII-EF-MCS). To produce a mammalian expression vector for the FLAG-tagged LOK (WT or active form), we subcloned a cDNA encoding full-length (amino acids 1–968) and kinase domain (amino acids 1–348) of human LOK together with a double-stranded oligonucleotide encoding FLAG epitope into pcDNA3.1 or a lentivirus vector (CSII-EF-MCS). All constructs were verified by sequencing.

### RNA interference and gene introduction using a lentivirus

iRNA-mediated interference was used to suppress mouse Rap1a/b and FLNa/b expression. A 19-nucleotide Rap1a/b (Rap1a: 5′- GAATGGCCAAGGGTTTGCA -3′, Rap1b: 5′- AGACACTGATGATGTTCCA -3′) or FLNa/b (FLNa: 5′- GGGCTATCGTGTCACCTAT -3′, FLNb: 5′- CCACAAAACCCGGAGAATA -3′)-specific sense iRNA sequence (5′–3′) or a scrambled control iRNA sequence was introduced into BAF cells using a lentivirus vector with or without GFP (a gift from Dr H. Miyoshi, RIKEN) containing the iRNA construct under control of the H1 promoter cassette. After infection, GFP-expressing cells were collected by cell sorting^36^.

### Immunoprecipitation and immunoblot analyses

COS cells, BAF cells or mouse T lymphocytes were lysed in buffer (1% Nonidet P-40, 150 mM NaCl, 25 mM Tris-HCl (pH 7.4), 10% glycerol, 2 mM MgCl_2_, 1 mM phenylmethylsulfonylfluoride, 1 mM leupeptin and 0.1 mM aprotinin). For membrane fractions, the cells were washed in RPMI and resuspended on ice in a hypotonic buffer. Cells were sheared, and nuclei and unbroken cells were removed using low-speed centrifugation. The remaining supernatant was recentrifuged, and the cytosolic fraction (supernatant) was removed. The remaining pellet (membrane fraction) was washed twice with hypotonic buffer and finally resuspended on ice in lysis buffer^44^. The lysates were precleared at 4 °C for 1 h with protein G-Sepharose 4B (GE Healthcare). Precleared lysates were immunoprecipitated with antibodies and protein G-Sepharose4B. The beads were washed four times with lysis buffer. Cell lysates or immunoprecipitates were subjected to immunoblotting^3^.

### Assessment of activation epitopes by monoclonal antibody staining

For KIM127 and MEM148 staining, cells were washed with binding buffer (10 mM HEPES, 150 mM NaCl, 2 mg m^−1^ BSA, 1 mM CaCl_2_ and 1 mM MgCl_2_ in HBSS), resuspended in 60 μl of the binding buffer and incubated for 30 min at 37 °C with 10 μg ml^−1^ of each monoclonal antibody in the presence or absence of 0.5 μM CXCL12. The mean fluorescence intensities were measured using a Gallios flow cytometry (Beckman Coulter)^43^.

### Pull-down assays

Rap1-GTP was pulled down with a GST-RBD of RalGDS fusion protein^45^. Briefly, 10^7^ cells were lysed in ice-cold lysis buffer (1% Triton X-100, 50 mM Tris-HCl (pH 7.5), 100 mM NaCl, 10 mM MgCl_2_, 1 mM phenylmethylsulfonyl fluoride, 1 mM leupeptin and 0.5 mM aprotinin) and incubated for 1 h at 4 °C with GST-fusion proteins coupled to glutathione agarose beads. The beads were washed three times with lysis buffer and subjected to western blot analysis using an anti-Rap1 antibody. Western blotting of total cell lysates (5 × 10^4^ cells) was also performed.

RhoA activation was also measured by pull-down assay using Rhotekin RBD-GST fusion protein. Cells stimulated with CXCL12 were lysed in a lysis buffer (25 mM HEPES (pH 7.5), 150 mM NaCl, 1% NP-40, 10 mM MgCl_2_, 1 mM EDTA, 10% glycerol, 22 μM leupeptin, 10 μg ml^−1^ aprotinin and 1 mM Na_3_VO_4_), incubated at 4 °C for 1 h with GST-Rhotekin-RBD immobilized on glutathione–Sepharose beads, and then washed three times with lysis buffer. The bound protein and total cell lysates were subjected to SDS–PAGE and immunoblotting with anti-RhoA antibody (Cell Signaling).

### *In vitro* kinase assay

Cells expressing FLAG-LOK or T_EM_ cells were lysed in lysis buffer (20 mM Tris-HCl pH 8.0, 150 mM NaCl, 1% NP-40, 1 mM phenylmethylsulphonyl fluoride, 10 μg ml^−1^ aprotinin and 1 mM Na_3_VO_4_). After removing insoluble debris with centrifugation, cell extracts were immunoprecipitated with anti-FLAG antibody or anti-LOK antibody. Immunoprecipitates were incubated in a kinase buffer (50 mM Tris-HCl (pH 7.2), 10 mM MgCl_2_, 10 mM MnCl_2_, 370 kBq γ-^32^P-ATP) containing 3 μg of myelin basic protein at 30 °C for 30 min (ref. 38). The samples were subjected to SDS–PAGE, and radioactivity was detected on a BAS-1800 image analyser (Fuji Film).

### Flow-adhesion assay

The human endothelial cell line LS12 was introduced with mouse ICAM-1, P-selectin, ICAM-1 or MadCAM-1. They were cultured on fibronectin-coated disks 1 day before the experiment and were pre-treated with TNFα for the last 6 h (ref. 4). In some experiments, disks were directly coated with CD300LG, GyCAM-1 or MAdCAM-1-IgG (10 μg ml^−1^ in PBS)^21^,^46^. These disks were incubated with or without chemokine (CXCL12, CCL21 or CXCL10, 0.5 μM) before placement in the flow chamber (FCS2; Bioptechs). Shear stress was generated using an automated syringe pump (Harvard Apparatus). Lymphocytes (1 × 10^6^ cells ml^−1^) or BAF/L-selectin/LFA-1 cells (1 × 10^6^ cells ml^−1^) suspended in pre-warmed RPMI1640 medium containing 10% FCS and 1 mM HEPES were infused into the flow chamber at 2 dyne cm^−2^ at 37 °C. In some experiments, cells were treated with the indicated antibodies. Phase-contrast images in a 0.32-mm^2^ microscopic field were recorded with an Olympus Plan Fluor DL × 10/0.3 numeric aperture objective lens at 3.3-ms intervals, and frame-by-frame displacements and velocities of lymphocyte movements were calculated by automatically tracking individual cells using the MetaMorph software (Molecular Devices). Interactions with each LS12 cell, PNAd or MadCAM-1 were categorized depending on dwell time: rolling; transient adhesion (0.5–10 s); and stable arrest (more than 10 s). The frequencies of cells exhibiting rolling, transient adhesion and stable arrest per minute are shown.

### Generation of recombinant CD300LG or GlyCAM-1 and MadCAM-1

CHO cells stably expressing human C2GnT, human FucTVII and human LSST (A5 cells) were transfected with pcDNA6 (Invitrogen) containing a cDNA encoding human C1GnT. The resultant line was named A5-Core1. These cells were transiently transfected with expression plasmids containing cDNA encoding CD300LG-Fc or GlyCAM-1-Fc using the Escort V lipofection reagent (Sigma-Aldrich). Chimeric proteins were purified from the culture supernatant using a protein A column. The cDNA encoding the extracellular region of rat MAdCAM-1 was amplified using PCR and inserted into pcDNAI-IgG to generate pcDNAI-MAdCAM1-IgG, which was co-transfected with pRSV-Neo into 293 cells using Lipofectin. Transfected cells were selected in medium containing 600 μg ml^−1^ of G418. Stable transfectants were cultured at a large scale in serum-free medium (ASF; Ajinomoto), and the supernatant of the culture was precipitated with 50% ammonium sulfate. After the precipitates were dissolved in and dialysed against PBS, chimeric protein was purified using HiTrap protein G affinity columns (Pharmacia), as recommended by the manufacturer.

### Lymphocyte migration on ICAM-1

Migration on ICAM-1 was investigated using a ΔT dish (Bioptecs) with immobilized recombinant human or mouse ICAM-1Fc (0.5 μg ml^−1^), with or without chemokine^6^. Images were obtained every 15 s for 15 min at 37 °C using Olympus IX50 equipped with a heating stage for a ΔT dish. Image data were analysed using the MetaMorph software. In each field, 50 randomly selected cells were automatically tracked to measure the velocity and displacement from the starting point.

### Confocal microscopy and time-lapse imaging

Non-adherent cells incubated with or without chemokine were fixed in suspension and immobilized on poly-L-lysine-coated slides before staining. Confocal images (TCS SP8, Leica) were obtained using a × 63 objective lens^36^. Time-lapse confocal images were also obtained in multitrack mode. Line profiles of the confocal images were obtained with the ImagePro software (MediaCybernetics).

### Histological examination

Colon sections were fixed in 10% buffered formalin and embedded in paraffin. Paraffin-embedded colon sections were cut (6 μm), stained with haematoxylin and eosin and examined on an Olympus IX51 light microscope equipped with a CCD (charge-coupled device) camera. Tissues were graded semiquantitatively. Histological grades were assigned in a blinded manner on a scale of 0–5, as follows: grade 0, no changes observed; grade 1, minimal scattered mucosal inflammatory cell infiltrates, with or without minimal epithelial hyperplasia; grade 2, mild scattered to diffuse inflammatory cell infiltrates, sometimes extending into the submucosa and associated with erosions, with mild to moderate epithelial hyperplasia and mild to moderate mucin depletion from goblet cells; grade 3, moderate inflammatory cell infiltrates that were sometimes transmural, with moderate to severe epithelial hyperplasia and mucin depletion; grade 4, marked inflammatory cell infiltrates that were often transmural and associated with crypt abscesses or occasional ulceration, with marked epithelial hyperplasia, mucin depletion; and grade 5, marked transmural inflammation with severe ulceration or loss of intestinal glands.

### Isolation of the colon LP

Intestines were opened longitudinally and washed in PBS to remove faeces. Intestines were placed in HBSS with 5 mM EDTA and incubated at 37 °C for 20 min in a shaking water bath (110 min^−1^). Intestines were poured into a Petri dish, rubbed against a paper towel to remove the epithelium, cut into small pieces in RPMI 1640 containing 4% FCS and incubated in RPMI 1640 containing 4% FCS, 1 mg ml^−1^ collagenase D (Roche), 0.5 mg ml^−1^ dispase (Invitrogen) and 40 μg ml^−l^ DNase I (Roche) for 60 min at 37 °C in a shaking water bath (110 min^−1^). The digested tissues were centrifuged and resuspended in 5 ml of 40% Percoll (GE Healthcare), and then overlaid on 2.5 ml of 80% Percoll in a 15-ml tube. Percoll gradient separation was performed by centrifugation at 780*g* for 20 min at room temperature. The LP lymphocytes were collected at the interface of the Percoll gradient and washed with PBS containing 2% FCS.

### Homing assay

Purified or cultured T cells were labelled with 1 μM 5, 6-carboxyfluorescein diacetate (CFSE, Invitrogen) and 10 μM (5-(and-6))((4-chloromethyl)benzoyl) amino) tetramethylrhodamine) (CMTMR, Invitrogen). An equal number of labelled control and Rap1a/b or FLNa-deficient T cells (1–5 × 10^6^) was injected intravenously into a normal C57BL/6 mouse. After 1 h, inguinal, mesentric and axillary LN cells, splenocytes and peripheral blood mononuclear cells were analysed using flow cytometry^47^. Reversal of fluorescent dyes yielded the same results. To examine the homing of T_EM_ ability to the colon, f/f or −/− T_EM_ cells (5 × 10^6^) were injected intravenously into a normal C57BL/6 mouse. After 10 h, the colon LP of the recipient mouse was analysed using flow cytometry.

### Transwell assay

Purified T cells were added to uncoated or ICAM-1-coated transwell insert with 3-μm-diameter pores (KURABO) at 2 × 10^6^ cells ml^−1^ in 500 μl of 1% FCS RPMI medium. CCL21 was added to RPMI medium (500 μl) in the lower chamber^37^. Migration was carried out for 1 h at 37 °C. Transwell inserts were removed, and the cells transmigrated into the lower chamber were counted. Cells that migrated into the lower chamber are shown as a percentage of the total number.

### HEV interactions of lymphocytes

Wild-type or Rap1a/b-deficient T cells were labelled with CFSE or CMTMR. Fluorescently labelled cells (1 × 10^6^ cells) were injected intravenously into the different recipient mice. The behaviour of cells in the mLN was observed under a fluorescence microscope (IX80; Olympus) equipped with a CCD camera (ORCA flash or EMCCD; Hamamatsu Photonics) at a 1-s interval^4^. Image acquisition was performed using the Metamorph software. The interactions under continuous flow were categorized as follows: ‘rolling' if the cells stopped for less than 1 s and ‘arrest' if the cells attached to the venule for 1 s.

### Treg suppression assay

Naive (CD4^+^CD45RB^high^CD25^−^) and regulatory (CD4^+^CD45RB^low^CD25^+^) T cells were isolated from a single-cell suspension of splenocytes and LNs by FACS sorting. After sorting, naive T cells were labelled with CFSE, counted and adjusted to 5 × 10^5^ ml^−1^ in complete RPMI culture media. Unlabelled Tregs were adjusted to 2.5 × 10^5^ ml^−1^. Cells were then co-cultured in a round-bottom 96-well plate at a Treg:naive T cell ratio of 1:2 and 1:4. The cells were stimulated with 1 μg ml^−1^ of soluble anti-CD3 and 2 μg ml^−1^ of anti-CD28 antibodies. After 48 h, the cells were collected and proliferation of naive T cells was determined by CFSE dilution and flow cytometric analysis.

### Intracellular cytokine staining

CD4^+^ T cells recovered from culture were activated with phorbol myristate acetate (50 ng ml^−1^) and ionomycin (1 μg ml^−1^) for 4 h at 37 °C in the presence of brefeldin A (10 μg ml^−1^). After staining with PECy7-anti-CD4 (RM4-5), cells were fixed in 4% paraformaldehyde/PBS for 10 min, permeabilized with HBSS containing 0.5% saponin and 1% FCS and stained with FITC-anti-IFNγ (XMG1.1), phycoerythrin -anti-IL-17A (TC11-18H10) and antigen-presenting cell-anti-IL-4 (11B11) for 30 min. Cells were then washed twice with HBSS containing 0.5% saponin and 1% FCS, resuspended in HBSS containing 1% FCS and subjected to flow cytometry.

### Statistical analysis

Statistical analysis was performed using analysis of variance (ANOVA) followed by the two-tailed multiple *t*-test with Bonferroni correction (Figs 1a,c and 4c left, 4d left, 4e left, 6b, 6c, 6e, 6f, 7i, 8a, 8c and 8e, and Supplementary Figs 1a,2d,3b and 6a–d) and two-tailed Student's *t*-test (Figs 1b,d,e and 2a–d, Figs 3a,b,e and 4c right, 4d right, 4e right, 4f, 6g, 6h, 8b and 8d and Supplementary Figs 1d, 4a and 4b). *P* values less than 0.05 were considered significant.

## Additional information

**How to cite this article:** Ishihara, S. *et al*. Dual functions of Rap1 are crucial for T-cell homeostasis and prevention of spontaneous colitis. *Nat. Commun.* 6:8982 doi: 10.1038/ncomms9982 (2015).

## Supplementary Material

Supplementary Figures and Supplementary Tables Supplementary Figures 1-8 and Supplementary Tables 1-3

Supplementary Movie 1 Rolling of control cells on the endothelium

Supplementary Movie 2 Rolling of Rap1*KD* cells on the endothelium

Supplementary Movie 3 Rolling of Rap1^f/f^ T_N_ cells on LS12

Supplementary Movie 4 Rolling of Rap1^-/-^ T_N_ cells on LS12

Supplementary Movie 5 Blebbing of control cells.

Supplementary Movie 6 Blebbing of Rap1*KD* cells

Supplementary Movie 7 Rolling of Rap1^f/f^ T_EM_ cells on MadCAM-1

Supplementary Movie 8 Rolling of Rap1^-/-^ T_EM_ cells on MadCAM-1

Supplementary Movie 9 Rolling of Rap1*KD* cells infected with control lentivirus

Supplementary Movie 10 Rolling of Rap1*KD* cells infected with active LOK-lentivirus

Supplementary Movie 11 Blebbing of control Rap1^-/-^ T_EM_ cells

Supplementary Movie 12 Blebbing of T567E ezrin Rap1^-/-^ T_EM_ cells

## References

[b1] Springer T. A. Traffic signals for lymphocyte recirculation and leukocyte emigration: the multistep paradigm. Cell 76, 301–314 (1994). 7507411 10.1016/0092-8674(94)90337-9

[b2] Miyasaka M. & Tanaka T. Lymphocyte trafficking across high endothelial venules: dogmas and enigmas. Nat. Rev. Immunol. 4, 360–370 (2004). 15122201 10.1038/nri1354

[b3] Katagiri K., Hattori M., Minato N. & Kinashi T. Rap1 functions as a key regulator of T-cell and antigen-presenting cell interactions and modulates T-cell responses. Mol. Cell Biol. 22, 1001–1015 (2002). 11809793 10.1128/MCB.22.4.1001-1015.2002 PMC134636

[b4] Ebisuno Y. . Rap1 controls lymphocyte adhesion cascade and interstitial migration within lymph nodes in RAPL-dependent and -independent manners. Blood 115, 804–814 (2009). 19965628 10.1182/blood-2009-03-211979

[b5] Katagiri K., Maeda A., Shimonaka M. & Kinashi T. RAPL, a Rap1-binding molecule that mediates Rap1-induced adhesion through spatial regulation of LFA-1. Nat. Immunol. 4, 741–748 (2003). 12845325 10.1038/ni950

[b6] Katagiri K. . Mst1 controls lymphocyte trafficking and interstitial motility within lymph nodes. EMBO J. 28, 1319–1331 (2009). 19339990 10.1038/emboj.2009.82 PMC2683056

[b7] Hogg N., Patzak I. & Willenbrock F. The insider's guide to leukocyte integrin signalling and function. Nat. Rev. Immunol. 11, 416–426 (2011). 21597477 10.1038/nri2986

[b8] Kiema T. . The molecular basis of filamin binding to integrins and competition with talin. Mol. Cell 21, 337–347 (2006). 16455489 10.1016/j.molcel.2006.01.011

[b9] Sharma C. P., Ezzell R. M. & Arnaout M. A. Direct interaction of filamin (ABP-280) with the beta 2-integrin subunit CD18. J. Immunol. 154, 3461–3470 (1995). 7534799

[b10] Gawecka J. E., Griffiths G. S., Ek-Rylander B., Ramos J. W. & Matter M. L. R-Ras regulates migration through an interaction with filamin A in melanoma cells. PLoS ONE 5, e11269 (2010). 20585650 10.1371/journal.pone.0011269 PMC2890414

[b11] Kurmaeva E. . T cell-associated α_4_β_7_ but not α_4_β_1_ integrin is required for the induction and perpetuation of chronic colitis. Mucosal Immunol. 7, 1354–1365 (2014). 24717354 10.1038/mi.2014.22 PMC4417258

[b12] Yu Y. . Structural specializations of α_4_β_7_, an integrin that mediates rolling adhesion. J. Cell Biol. 196, 131–146 (2012). 22232704 10.1083/jcb.201110023 PMC3255974

[b13] Firrell J. C. & Lipowsky H. H. Leukocyte margination and deformation in mesenteric venules of rat. Am. J. Physiol. 256, H1667–H1674 (1989). 2735435 10.1152/ajpheart.1989.256.6.H1667

[b14] Lek H. S. . The spontaneously adhesive leukocyte function-associated antigen-1 (LFA-1) integrin in effector T cells mediates rapid actin-and calmodulin-dependent adhesion strengthening to ligand under shear flow. J. Biol. Chem. 288, 14698–14708 (2013). 23585567 10.1074/jbc.M112.430918 PMC3663495

[b15] Ghandour H., Cullere X., Alvarez A., Luscinskas F. W. & Mayadas T. N. Essential role for Rap1 GTPase and its guanine exchange factor CalDAG-GEFI in LFA-1 but not VLA-4 integrin mediated human T-cell adhesion. Blood 110, 3682–3690 (2007). 17702895 10.1182/blood-2007-03-077628 PMC2077316

[b16] Sun H. . Distinct chemokine signaling regulates integrin ligand specificity to dictate tissue-specific lymphocyte homing. Dev. Cell 30, 61–70 (2014). 24954024 10.1016/j.devcel.2014.05.002

[b17] Parameswaran N. & Gupta N. Re-defining ERM function in lymphocyte activation and migration. Immunol. Rev. 256, 63–79 (2013). 24117813 10.1111/imr.12104

[b18] Liu Y. . Constitutively active ezrin increases membrane tension, slows migration, and impedes endothelial transmigration of lymphocytes *in vivo* in mice. Blood 119, 445–453 (2011). 22106344 10.1182/blood-2011-07-368860 PMC3257010

[b19] Belkina N. V., Liu Y., Hao J. J., Karasuyama H. & Shaw S. LOK is a major ERM kinase in resting lymphocytes and regulates cytoskeletal rearrangement through ERM phosphorylation. Proc. Natl Acad. Sci. USA 106, 4707–4712 (2009). 19255442 10.1073/pnas.0805963106 PMC2660762

[b20] Dupuy A. G. . Novel Rap1 dominant-negative mutants interfere selectively with C3G and Epac. Oncogene 24, 4509–4520 (2005). 15856025 10.1038/sj.onc.1208647

[b21] Umemoto E. . Nepmucin, a novel HEV sialomucin, mediates L-selectin-dependent lymphocyte rolling and promotes lymphocyte adhesion under flow. J. Exp. Med. 203, 1603–1614 (2006). 16754720 10.1084/jem.20052543 PMC2118321

[b22] Sundd P., Pospieszalska M. K. & Ley K. Neutrophil rolling at high shear: flattening, catch bond behavior, tethers and slings. Mol. Immunol. 55, 59–69 (2013). 23141302 10.1016/j.molimm.2012.10.025 PMC3601566

[b23] Dai J. & Sheetz M. P. Membrane tether formation from blebbing cells. Biophys. J. 77, 3363–3370 (1999). 10585959 10.1016/S0006-3495(99)77168-7 PMC1300608

[b24] Edmondson K. E., Denney W. S. & Diamond S. L. Neutrophil-bead collision assay: pharmacologically induced changes in membrane mechanics regulate the PSGL-1/P-selectin adhesion lifetime. Biophys. J. 89, 3603–3614 (2005). 16100264 10.1529/biophysj.105.066134 PMC1366853

[b25] Yago T. . Distinct molecular and cellular contributions to stabilizing selectin-mediated rolling under flow. J. Cell Biol. 158, 787–799 (2002). 12177042 10.1083/jcb.200204041 PMC2174028

[b26] Sundd P., Pospieszalska M. K., Cheung L. S., Konstantopoulos K. & Ley K. Biomechanics of leukocyte rolling. Biorheology 48, 1–35 (2011). 21515934 10.3233/BIR-2011-0579 PMC3103268

[b27] Paluch E. K. & Raz E. The role and regulation of blebs in cell migration. Curr. Opin. Cell Biol. 25, 582–590 (2013). 23786923 10.1016/j.ceb.2013.05.005 PMC3989058

[b28] Tlaskalova-Hogenova H. . Involvement of innate immunity in the development of inflammatory and autoimmune diseases. Ann. N Y Acad. Sci. 1051, 787–798 (2005). 16127016 10.1196/annals.1361.122

[b29] Schepis A., Sepich D. & Nelson W. J. αE-catenin regulates cell-cell adhesion and membrane blebbing during zebrafish epiboly. Development 139, 537–546 (2012). 22190637 10.1242/dev.073932 PMC3252354

[b30] Sliogeryte K., Thorpe S. D., Lee D. A., Botto L. & Knight M. M. Stem cell differentiation increases membrane-actin adhesion regulating cell blebability, migration and mechanics. Sci. Rep. 4, 7307 (2014). 25471686 10.1038/srep07307 PMC4255193

[b31] Martinelli S. . Ezrin/Radixin/Moesin proteins and flotillins cooperate to promote uropod formation in T cells. Front. Immunol. 4, 84 (2013). 23579783 10.3389/fimmu.2013.00084 PMC3619129

[b32] Brown M. J. . Chemokine stimulation of human peripheral blood T lymphocytes induces rapid dephosphorylation of ERM proteins, which facilitates loss of microvilli and polarization. Blood 102, 3890–3899 (2003). 12907449 10.1182/blood-2002-12-3807

[b33] del Pozo M. A., Vicente-Manzanares M., Tejedor R., Serrador J. M. & Sanchez-Madrid F. Rho GTPases control migration and polarization of adhesion molecules and cytoskeletal ERM components in T lymphocytes. Eur. J. Immunol. 29, 3609–3620 (1999). 10556816 10.1002/(SICI)1521-4141(199911)29:11<3609::AID-IMMU3609>3.0.CO;2-S

[b34] Takala H. . β_2_ integrin phosphorylation on Thr758 acts as a molecular switch to regulate 14-3-3 and filamin binding. Blood 112, 1853–1862 (2008). 18550856 10.1182/blood-2007-12-127795

[b35] Kawabe T. . Homeostatic proliferation of naive CD4^+^ T cells in mesenteric lymph nodes generates gut-tropic Th17 cells. J. Immunol. 190, 5788–5798 (2013). 23610141 10.4049/jimmunol.1203111

[b36] Katagiri K., Imamura M. & Kinashi T. Spatiotemporal regulation of the kinase Mst1 by binding protein RAPL is critical for lymphocyte polarity and adhesion. Nat. Immunol. 7, 919–928 (2006). 16892067 10.1038/ni1374

[b37] Nishikimi A. . Rab13 acts downstream of the kinase Mst1 to deliver the integrin LFA-1 to the cell surface for lymphocyte trafficking. Sci. Signal. 7, ra72 (2014). 25074980 10.1126/scisignal.2005199

[b38] Endo J. . Deficiency of a STE20/PAK family kinase LOK leads to the acceleration of LFA-1 clustering and cell adhesion of activated lymphocytes. FEBS Lett. 468, 234–238 (2000). 10692593 10.1016/s0014-5793(00)01219-9

[b39] Teo G. S. . Mesenchymal stem cells transmigrate between and directly through tumor necrosis factor-α-activated endothelial cells via both leukocyte-like and novel mechanisms. Stem Cells 30, 2472–2486 (2012). 22887987 10.1002/stem.1198 PMC3479371

[b40] Popowicz G. M., Schleicher M., Noegel A. A. & Holak T. A. Filamins: promiscuous organizers of the cytoskeleton. Trends Biochem. Sci. 31, 411–419 (2006). 16781869 10.1016/j.tibs.2006.05.006

[b41] Pentikainen U. & Ylanne J. The regulation mechanism for the auto-inhibition of binding of human filamin A to integrin. J. Mol. Biol. 393, 644–657 (2009). 19699211 10.1016/j.jmb.2009.08.035

[b42] Scarpa M. . Inflammatory colonic carcinogenesis: a review on pathogenesis and immunosurveillance mechanisms in ulcerative colitis. World J. Gastroenterol. 20, 6774–6785 (2014). 24944468 10.3748/wjg.v20.i22.6774 PMC4051917

[b43] Katagiri K. . Rap1 is a potent activation signal for leukocyte function-associated antigen 1 distinct from protein kinase C and phosphatidylinositol-3-OH kinase. Mol. Cell Biol. 20, 1956–1969 (2000). 10688643 10.1128/mcb.20.6.1956-1969.2000 PMC110813

[b44] Kliche S. . CCR7-mediated LFA-1 functions in T cells are regulated by 2 independent ADAP/SKAP55 modules. Blood 119, 777–785 (2012). 22117043 10.1182/blood-2011-06-362269

[b45] Katagiri K., Shimonaka M. & Kinashi T. Rap1-mediated lymphocyte function-associated antigen-1 activation by the T cell antigen receptor is dependent on phospholipase C-γ1. J. Biol. Chem. 279, 11875–11881 (2004). 14702343 10.1074/jbc.M310717200

[b46] Kobayashi M. . GlcNAc6ST-1-mediated decoration of MAdCAM-1 protein with L-selectin ligand carbohydrates directs disease activity of ulcerative colitis. Inflamm. Bowel Dis. 15, 697–706 (2009). 19067429 10.1002/ibd.20827 PMC2696616

[b47] Katagiri K. . Crucial functions of the Rap1 effector molecule RAPL in lymphocyte and dendritic cell trafficking. Nat. Immunol. 5, 1045–1051 (2004). 15361866 10.1038/ni1111

