# Supplementary material for: Dual functions of Rap1 are crucial for T-cell homeostasis and prevention of spontaneous colitis
Source: Nat Commun. 2015 Dec 4;6:8982. doi: 10.1038/ncomms9982 (PMC4686857; doi:10.1038/ncomms9982)
Supplement: Supplementary Figures and Supplementary Tables — Supplementary Figures 1-8 and Supplementary Tables 1-3 [file ncomms9982-s1.pdf]

Supplementary Figure 1

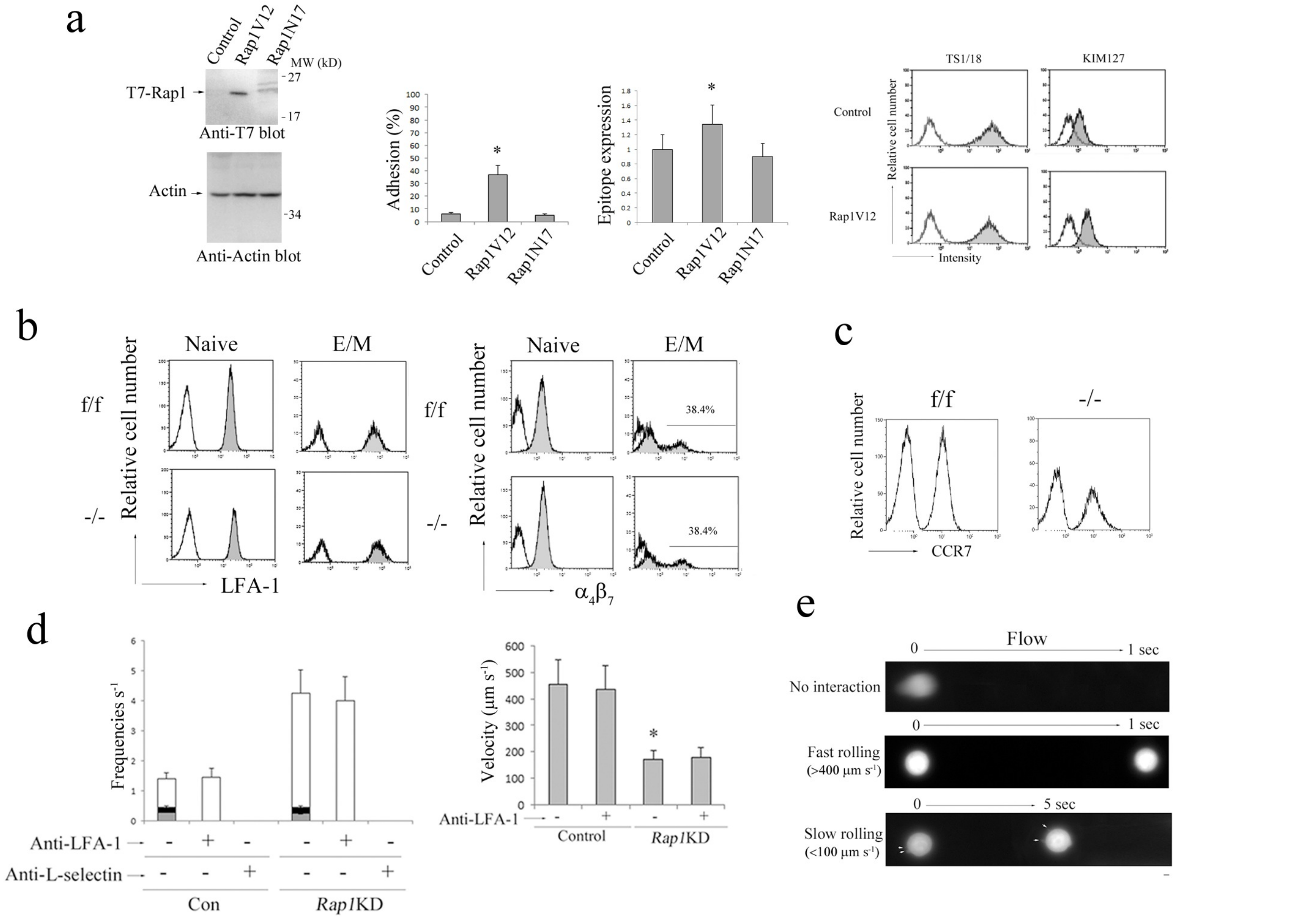

Supplementary Figure 1. Flow-cytometry profiles and membrane tethers in Rap1-deficient cells.

(a) (Left) Total cell lysates from *Rap1*KD cells introduced with control, T7-tagged Rap1V12 or Rap1N17 were immunoblotted with anti-T7. Actin was used as a loading control. A representative blot of three experiments is shown. (Center) LFA-1/ICAM-1-dependent adhesion of Rap1V12 or Rap1N17-expressing *Rap1*KD cells under static condition was measured by detachment assay. After the cells were incubated on the ICAM-1 for 10 min, shear flow (2 dyn cm^{-2}) was applied, and shear-resistant firmly adhered cells were counted. Data represent the means ± s.e.m. of triplicate experiments (n = 50 for each group). *P < 0.001, compared with control *Rap1*KD cells. (Right) Epitope expression of mAb KIM127on Rap1V12 or Rap1N17-expressing *Rap1*KD cells. Data are normalized against LFA-1 expression detected by TS1/18. Data represent the means ± s.e.m. of triplicate experiments. *P < 0.02, compared with control *Rap1*KD cells. Representative flow-cytometry profiles of TS1/18 and KIM127 expression in control or Rap1V12-expressing *Rap1*KD cells are shown in the right side end; baseline (open histogram). **(b)** Representative flow-cytometry profiles of LFA-1 (left) and $\alpha_4\beta_7$ (right) expression of naïve and effector/memory (E/M) T cells from mLNs of f/f and -/- mice. **(c)** Representative flow cytometry profiles of CCR7 expression of naïve T cells from mLN of f/f and -/- mice. **(d)** (Left) Effects of anti–LFA-1 or L-selectin on the interaction frequencies of control and *Rap1*KD T cells on LS12 endothelial cells in the absence of CXCL12. Cells were pretreated with or without anti–LFA-1or L-selectin, and then perfused at 2 dyne cm^{-2} on LS12 monolayers. Data represent the means ± s.e.m. of triplicate experiments (n = 150 for each group). (Right) Rolling velocities of control and *Rap1*KD cells on LS12 endothelial cells in the absence of CXCL12. Cells were pretreated with or without anti–LFA-1. Data represent the means ± s.e.m. of triplicate experiments (n = 150 for each group). *P < 0.001, compared with control cells. **(e)** Morphology of rolling control and *Rap1*KD cells expressing GFP on the endothelium were observed using a 63× objective lens under shear flow. Sequential images of a representative control cell exhibiting no interaction with the endothelium or fast rolling (>400 μm s^{-1}), and *Rap1*KD cell exhibiting slow rolling (<100 μm s^{-1}) are provided. Scale bars, 5 μm. Arrows show tethers. Arrows indicate blebs. Scale bar, 5 μm.

Supplementary Figure 2

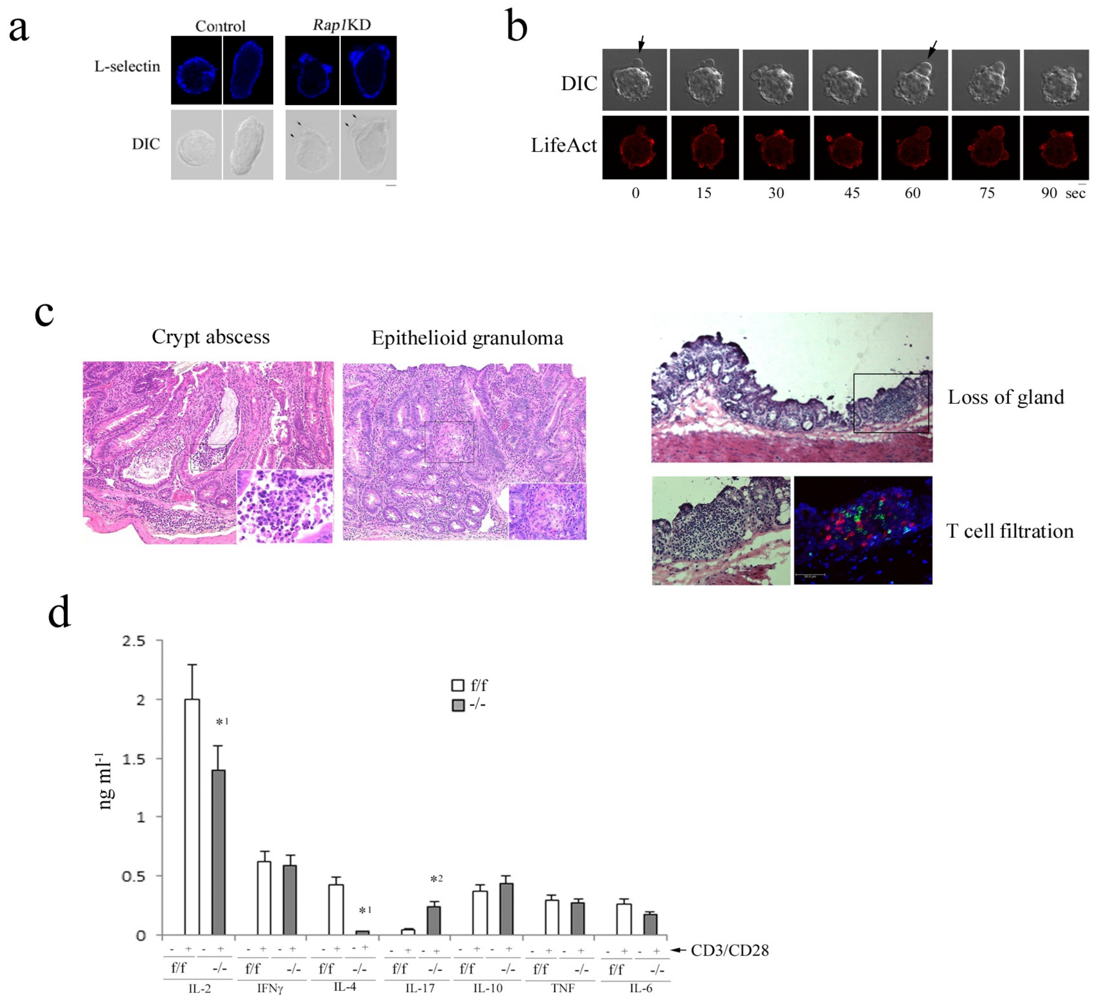

Supplementary Figure 2. Blebs and cytokine production in Rap1-deficient cells.

(a) Cellular localization of L-selectin in control or *Rap1*-knockdown BAF/L-selectin/LFA-1 cells. Two representative cells are shown. DIC, differential interference contrast. Scale bar, 5 μm. Arrows indicate bleb-like protrusion.

(b) Representative confocal images of expansion and retraction of blebs in Rap1-deficient cells transfected with LifeAct-mCherry are shown. Digital images were taken every 5 sec. Scale bar, 5 μm.

(c) Paraffin-embedded sections of the colon from -/- mice were stained with hematoxylin and eosin. Representative low (40×) and high (200×) (region surrounded with a square in 40x image) magnification histological images of large intestine from -/- mice are shown. Colon section showing loss of gland was also stained with anti-CD3 (red) and anti-B220 (green).

(d) Cytokine production of f/f and -/- $CD4^+$ T_N cells. Primary naïve $CD4^+$ T cells from LNs and spleen of f/f (open bar) or -/- (closed bar) mice were unstimulated or stimulated with anti-CD3 and anti-CD28. Cytokines in supernatants were measured in triplicate 3 d after stimulation. Data represent the means ± s.e.m. of three independent experiments. $*^1P < 0.001$, $*^2P < 0.005$, relative to the corresponding wild-type littermate control.

Supplementary Figure 3

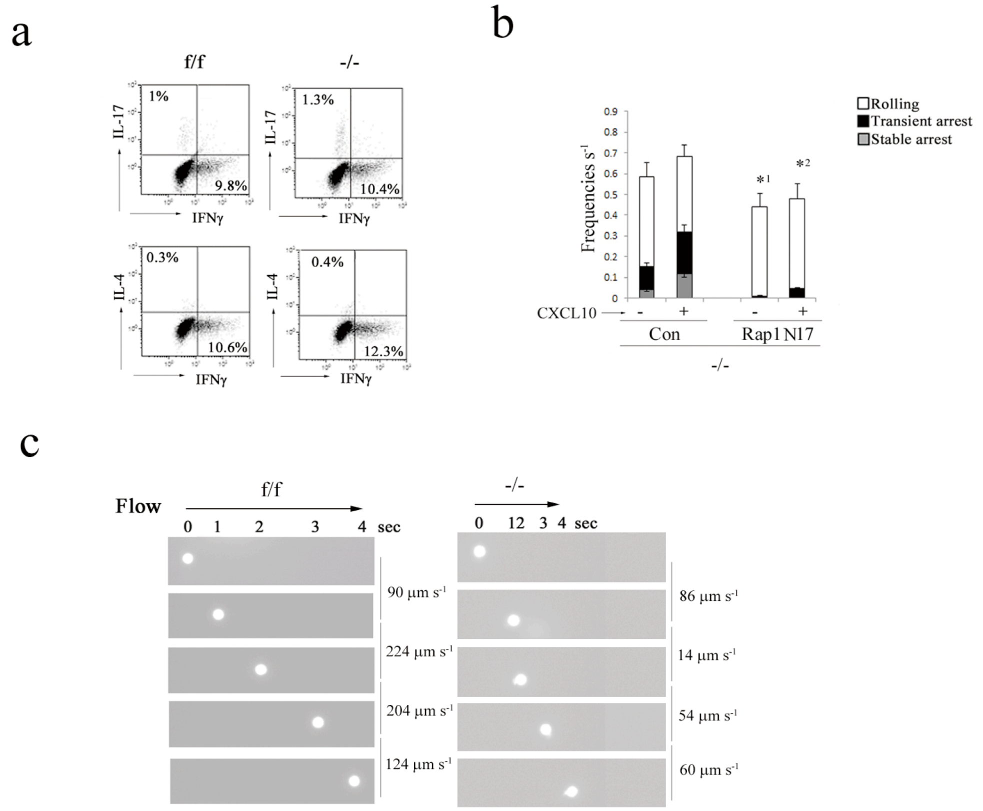

Supplementary Figure 3. Analysis of Rap1-deficient CD4$^+$ T$_{EM}$ cells.

(a) Cytokine profiles of f/f or -/- CD4$^+$ T$_{EM}$ cells. CD4$^+$ T$_N$ cells from f/f or -/- mice were cultured for 3 d under T$_H$17-polarizaing conditions, stimulated for 4 h with PMA plus ionomycin, and then subjected to flow cytometry to determine the frequency of T$_H$1 or T$_H$17, or T$_H$1 and T$_H$2, among CD4$^+$ cells based on their production of IFN-γ, IL-17, and IL-4. Data are representative of three independent experiments.

(b) -/- T$_{EM}$ cells infected with control or Rap1N17–encoding lentivirus that were differentiated under T$_H$17-polarizing conditions, perfused at 2 dyne cm^{-2} on plates immobilized with purified MadCAM-1 in the presence or absence of 100 nM CXCL10. Adhesive events of more than 100 cells were measured as in Fig. 1b. Data represent the means ± s.e.m. of three independent experiments. $*^1P < 0.01$, $*^2P < 0.03$, compared to the total frequency of control -/- T$_{EM}$ cells.

(c) Morphology of rolling f/f (left) and -/- (right) T$_{EM}$ cells labeled with CFSE on the MadCAM-1were observed using a 63× objective lens under shear flow. Sequential images of a representative f/f or -/-T$_{EM}$ cell at the indicated times are provided. Scale bars, 5 μm.

.

Supplementary Figure 4

a

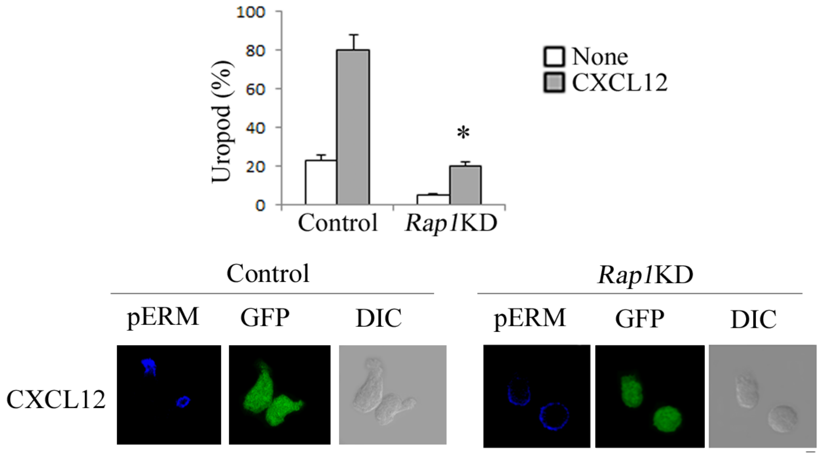

b

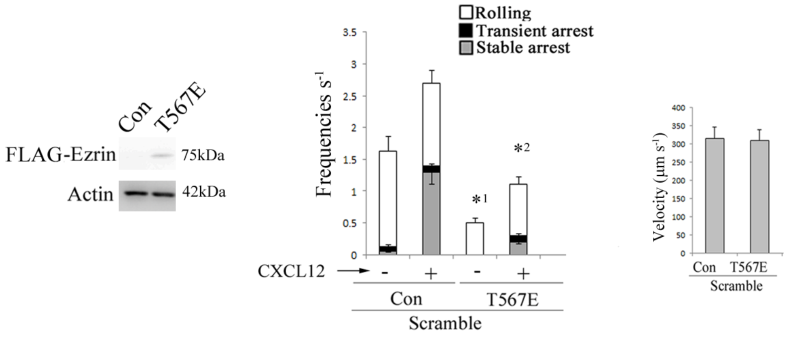

Supplementary Figure 4. Impaired uropod formation in *Rap1*KD cells in the presence of CXCL12 stimulation.
(a) (Upper) Control or *Rap1* KD cells were either unstimulated (open bar) or stimulated (closed bar) with 100 nM of CXCL12 for 10 min at 37°C. The frequency of cells exhibiting increases of more than 5-fold in phosphorylated ezrin and moesin intensity on the uropod, relative to the peak value of intensity on the side and rear, was measured in the absence or presence of CXCL12. Data represent the means ± s.e.m. of triplicate experiments (n = 40 cells of each group). *$P < 0.001$, relative to control cells. (Lower) Control or *Rap1*KD cells stimulated with 100 nM of CXCL12 were stained with anti-pERM. Two representative cells are shown. DIC, differential interference contrast. Scale bar, 5 μm.
(b) (Left) Western blots of total cell lysates from control (scramble) with control or lentivirus encoding ezrin mutant (T567E) are shown. Actin was used as a loading control. The blot shown is representative of four independent experiments. (Center) The adhesive events of scramble control cells or phosphomimetic mutant of ezrin (T567E)- expressing cells, with LS12 cells were measured as in Fig. 1a. Data represent the means ± s.e.m. of three independent experiments. $*^{1}P < 0.005$, $*^{2}P < 0.001$, relative to total frequencies of the corresponding control scramble cells. (Right) Rolling velocities of control cells or phosphomimetic mutant of ezrin (T567E)-expressing cells, on LS12 endothelial cells in the absence of CXCL12. Data represent the means ± s.e.m. of triplicate experiments (n = 150 per each group).

Supplementary Figure 5

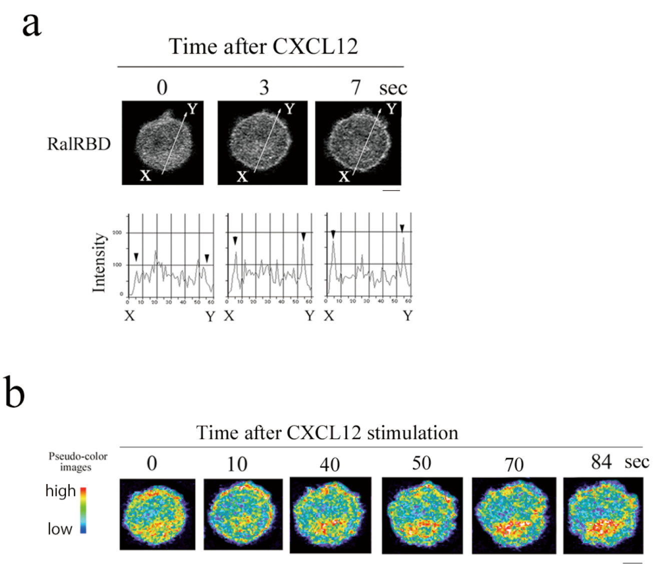

Supplementary Figure 5. Localization of Rap1-GTP after chemokine stimulation.

(a) (upper) Translocation of RalGDS-RBD-mcherry to the plasma membrane was observed at 3 sec after CXCL12 stimulation. A fusion protein of RalGDS-RBD and mCherry was expressed in wild-type cells, and its spatial redistribution was examined in response to CXCL12. The cell shown is representative of five experiments. Scale bar, 5 μm. (Lower) Line profiles indicate RalGDS-RBD-mcherry intensity along the arrows (X-Y) in the cell images.

(b) RalGDS-RBD-mcherry translocated to the perinuclear region at 70 sec after CXCL12 stimulation. Pseudo-color images of RalGDS-RBD-mcherry intensities of confocal images of the cells at the indicated times after CXCL12 stimulation are shown. Scale bar, 5 μm.

Supplementary Figure 6

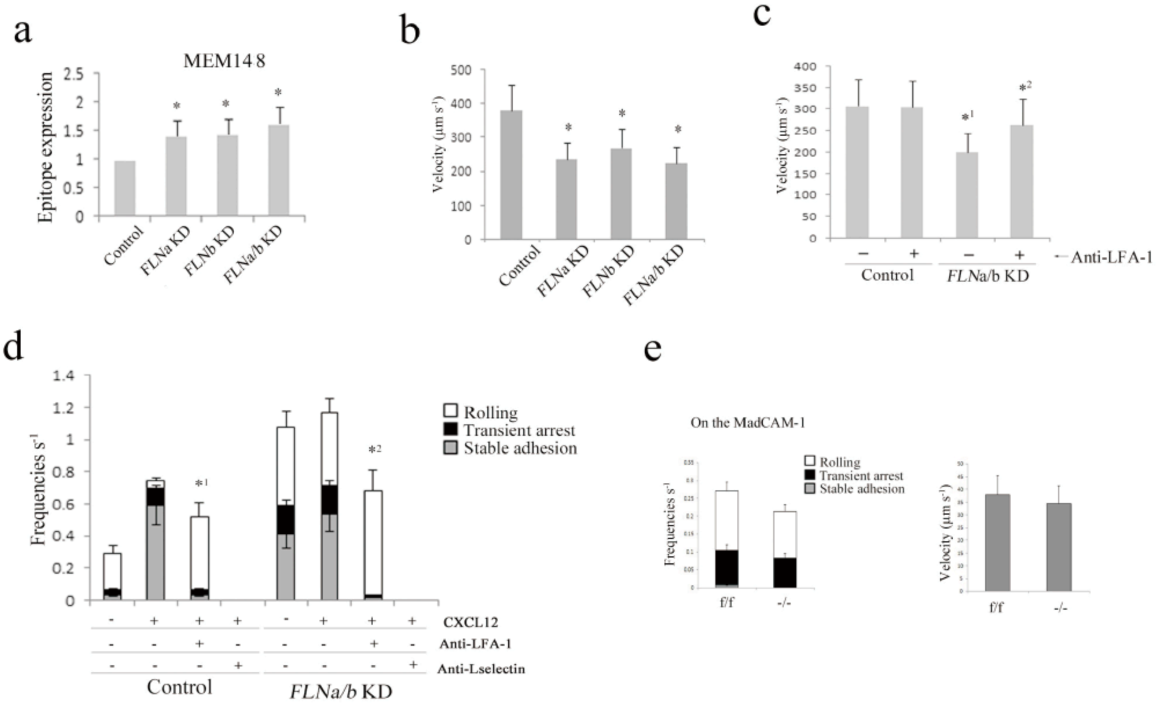

Supplementary Figure 6. Analysis of LFA-1 in *FLNa/b* knockdown cells.

(a) Epitope expression of MEM148 on control or *FLNa/b* KD cells. Data are normalized for LFA-1 expression detected by TS1/18. Data represent the means ± s.e.m. of triplicate experiments. **P* < 0.01, compared with control cells.

(b) Rolling velocities of control or *FLNa/ b* KD cells are shown. Data represent the means ± s.e.m. of triplicate experiments (n = 150 cells per each group). **P* < 0.01, relative to control cells.

(c) Effects of anti–LFA-1 on the rolling velocities of control or *FLNa/b* KD cells are shown. $*^1P < 0.02$, relative to control cells. $*^2P < 0.05$, relative to *FLNa/b* KD cells in the absence of anti–LFA-1.

(d) Control cell and *FLNa/b* KD cells perfused at 2 dyne/cm^2 on LS12 monolayers immobilized with or without 100 nM CXCL12 in the presence or absence of anti–LFA-1 or anti–L-selectin. Data represent the means ± s.e.m. of triplicate experiments (n = 150 cells per each group). $*^1P < 0.02$, $*^2P < 0.01$, relative to CXCL12-stimulated control or *FLNa/b* KD cells in the absence of anti–LFA-1.

(e) (left) The adhesive events of f/f and FLNa/b-deficient T cells on the MadCAM-1 in the absence of CCL21 under shear flow were measured as in Fig. 1b. Data represent the means ± s.e.m. of triplicate experiments (n = 100 cells per each group). (Right) Rolling velocities of f/f and FLNa/b-deficient T cells are shown. Data represent the means ± s.e.m. of triplicate experiments (n = 100 cells per each group).

Supplementary Figure 7

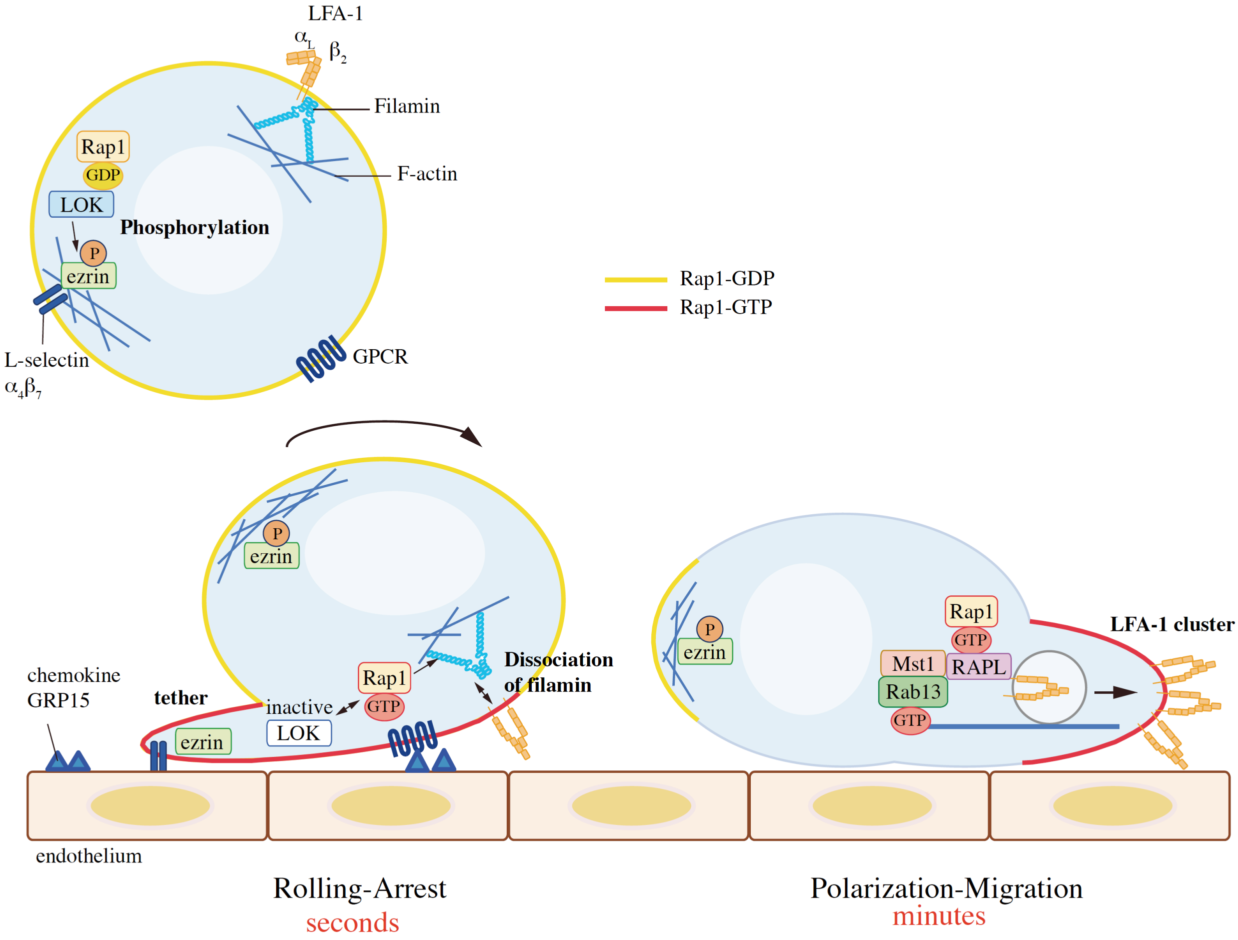

Supplementary Figure 7. Regulation of T-cell adhesion cascade by both GTP- and GDP-binding forms of Rap1.

Rap1-GDP in resting cells retains cell rigidity in blood vessels and limits rolling behaviors on the PNAd, MadCAM-1 and P-selectin through LOK activation and basal phosphorylation of ERM. Filamins associates with inactive bent conformation of LFA-1.

When rolling lymphocytes are exposed to chemokines present on the endothelium, chemokine signaling coupled with Gi proteins activates Rap1. Within seconds, Rap1-GDP converts into GTP-binding form, and 1) decreases in LOK activity and phosphorylation of ERM facilitate rolling behaviors of T_N and T_{EM} cells through the formation of tethers. At same time, 2) the association of filamins with Rap1-GTP induces its dissociation from β_2, which leads to the transition of external domains of LFA-1 from a bent conformation to an active extended one, leading to arrest of T_N cells on the HEV. In minutes, localization of Rap1-GTP at the perinuclear region and the leading edge induced cell polarization and Rab13-dependent delivery of LFA-1 to the leading edge through RAPL-Mst1 along actin cable.

Rap1-GTP is dispensable for adhesion of T_{EM} cells to the endothelium, although it promotes their LFA-1 and $\alpha_4\beta_7$-dependent adhesion. Therefore, Rap1-deficientcy in T_N cells impaired homing into secondary lymph nodes, but that in T_{EM} cells facilitated their homing into colon.

Supplementary Figure 8

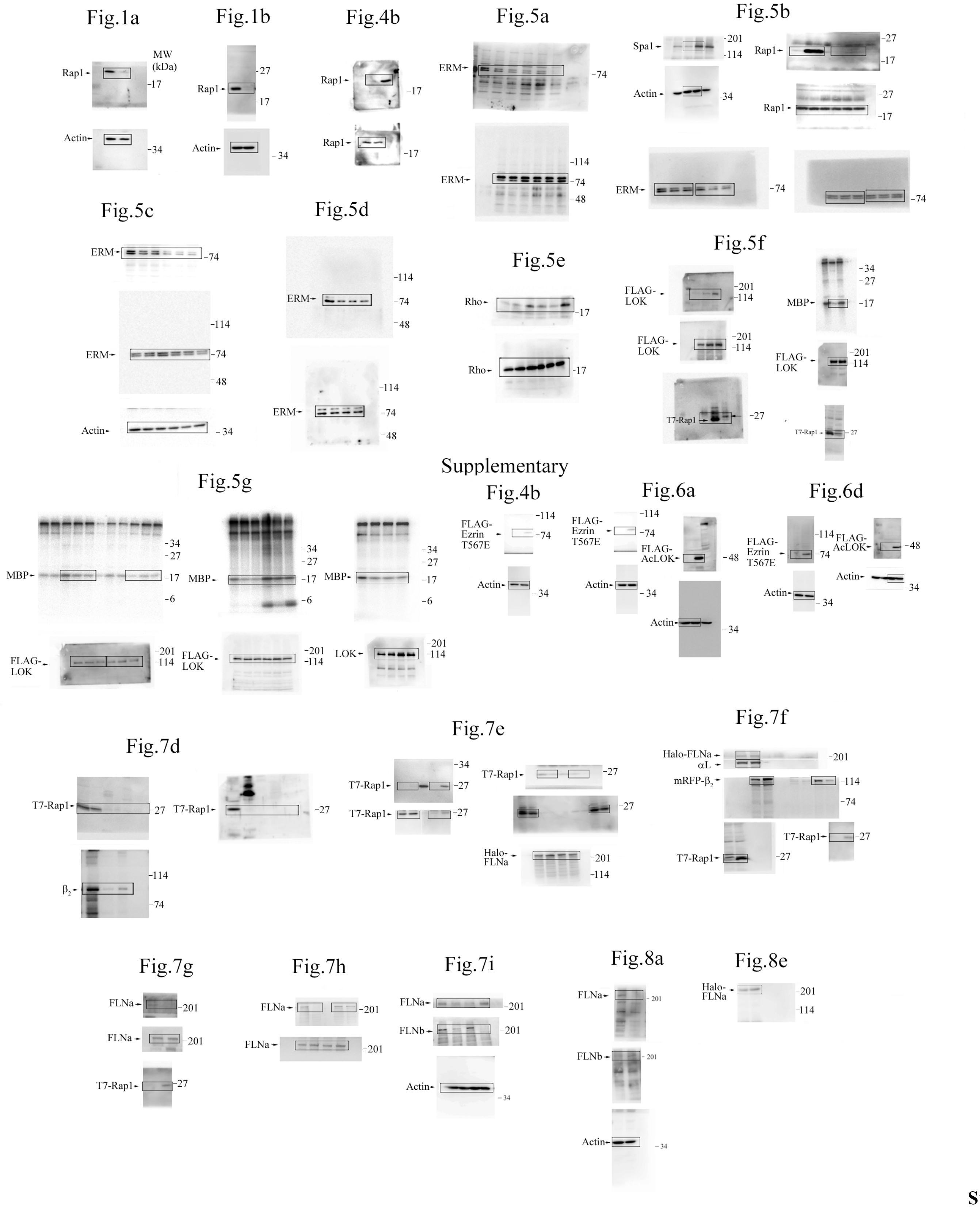

Su

pplementary Figure 8. Full blots for immunoblotting and GST pull down assay shown in Figures and Supplementary Figures.

Supplementary Table 1. Sequences of primers used for cloning.

Product	Forward primer (5' to 3')	Reverse primer (5' to 3')
FLNa (1-3)	ataggatccgggccaggcatcgagcccac	aragaattcccaggcccgtaccttctgat
FLNa (4-6)	ataggatccggccctgggctggagggcgg	atagaattcctcggccttcaccttactgg
FLNa (7-10)	atagaattcggccctggcctcagtcgcac	atagcggccgcatagcactggacaccggaag
FLNa (21)	ataggatccggccctggcctggagagagc	atagcggccgcaacagtgaggcggcgggcgt
β2 (cytoplasmic region)	aaaggatcctggaaggctctgatccacc	aacgaattcctaactctcagcaaacttgg
Lifeact-mCherry	gcggatccaccatgggtgtcgcagatttgatcaagaaattcgaaagcatctcaaaggaagaaggcggcagcggcatggtgagcaagggcgagga	ggaattctacttgtacagctcgtcca
FLAG-tagged ezrin	aaagcggccgcaccatggactacaaggatgacgatgacaagatgccgaaaccaatcaatgt	aaagcggccgcttacagggcctcgaactcgt
EGFP	gcgctagcgccaccatggtgagcaagggcg	ataagcttcttgtacagctcgtccatgc
RalGDS-RBD	gcggatccgcgctgccgctctacaacca	tactcgagttaggtccgcttcttgaggacaa
Venus	gtcagcggccgcaatggtgagcaagggcgaggagctg	gccggatcccttgtacagctcgtccatgccgag
Rap1	ctcggcatggacgagctgtacaagggatccggcatgcgtgagtacaagctagtggtc	gtcgagctcggatccctagagcagcagacatgatttctt
mTarquoise	ggctccggcatggtgagcaagggcgaggagctg	gactgcggccgcagatctttattaggatcccttgtacagctcgtccatgccgag
RAPL-RBD	gagaattcgccaccatgaatgtctgtaaacctgtggagga	ctcctcgcccttgctcaccatgccggagccagtttcattctcctttagcac
LOK (full length)	atgatatcaccatggcttttgccaatttccg	tagtcgacagaagcatccgcagaactg
LOK (1-348)	atgatatcaccatggcttttgccaatttccg	atgtcgacactcacctcagaggagttct

Restriction enzyme recognition sequences are underlined.

Supplementary Table 2. Sequences of oligonucleotides used for mutagenesis.

Oligonucleotide	Sequence (5' to 3')
Rap1 A17 sense	tcaggaggcgttgggaaggctgctctgacagttcagttt
Rap1 A17 antisense	aaactgaactgtcagagcagccttcccaacgcctcctga
Ezrin T576E sense	gagctgcggcagatccggcagggcaac
Ezrin T576E antisense	cttgtacttgtcccggcctt
FLNa D3 sense	aaccgcccggcaggcactcgggtta
FLNa D3 antisense	ggccctgggctggagggcggcgt

Supplementary Table 3. Sequences of oligonucleotides encoding FLAG (5’ to 3’).

Sense	tcgaggactacaaagacgatgacgacaagtaat
Antisense	ctagattacttgtcgtcatcgtctttgtagtcc
